# An experimental model for ovarian cancer: propagation of ovarian cancer initiating cells and generation of ovarian cancer organoids

**DOI:** 10.1186/s12885-022-10042-3

**Published:** 2022-09-10

**Authors:** Yu-An Chen, Chen-Yu Lu, Wen-Fang Cheng, Kuan-Ting Kuo, Chen-Wei Yu, Hon-Nerng Ho, Hsin-Fu Chen, Szu-Hua Pan

**Affiliations:** 1 Graduate Institute of Medical Genomics and Proteomics, College of Medicine, National Taiwan University, Taipei, 100 Taiwan; 2 Graduate Institute of Oncology, College of Medicine, National Taiwan University, Taipei, 100 Taiwan; 3 Department of Obstetrics and Gynecology, College of Medicine and the Hospital, National Taiwan University, Taipei, 100 Taiwan; 4 Department of Pathology and Graduate Institute of Pathology, College of Medicine, National Taiwan University, Taipei, 100 Taiwan; 5 Department of Pathology, College of Medicine and the Hospital, National Taiwan University, Taipei, 100 Taiwan; 6 Ph.D. Program in Translational Medicine, National Taiwan University and Academia Sinica, Taipei, 100 Taiwan; 7 Department of Statistics and Information Science, Fu Jen Catholic University, Taipei, 242 Taiwan; 8 Genome and Systems Biology Degree Program, Academia Sinica and National Taiwan University, Taipei, 106 Taiwan

**Keywords:** Ovarian cancer (OC), Cancer-initiating cells (CICs), Induced ovarian cancer-initiating cells (iOCICs), Organoid and drug screening

## Abstract

**Background:**

Ovarian cancer (OC) is the most lethal gynecological cancer due to the recurrence of drug-resistance. Cancer initiating cells (CICs) are proposed to be responsible for the aggressiveness of OC. The rarity and difficulty of in vitro long-term cultivation of CICs challenge the development of CIC-targeting therapeutics. Reprogramming cancer cells into induced cancer initiating cell (iCICs) could be an approach to solve these. Several inducible CICs have been acquired by activating the expression of stemness genes in different cancer cells. However, few reports have demonstrated the feasibility in OC.

**Methods:**

Patients with primary OC receiving surgery were enrolled. Tumor tissue were collected, and OCT4, SOX2, and NANOG expressions were assessed by immunohistochemistry (IHC) staining to investigate the association of stemness markers with overall survival (OS). An high-grade serous ovarian cancer (HGSOC) cell line, OVCAR-3 was reprogrammed by transducing Yamanaka four factors *OCT4, SOX2, KLF4* and *MYC* (*OSKM*) to establish an iOCIC model, iOVCAR-3-OSKM. CIC characteristics of iOVCAR-3-OSKM were evaluated by RT-PCR, sphere formation assay and animal experiments. Drug-resistance and migration ability were accessed by dye-efflux activity assay, MTT assay and migration assay. Gene profile was presented through RNA-sequencing. Lineage differentiation ability and organoid culture were determined by in vitro differentiation assays.

**Results:**

In OC patients, the co-expression of multiple stem-related transcription factors (*OCT4, SOX2*, and *NANOG*) was associated with worse OS. iOVCAR-3-OSKM cells generated by reprogramming successfully exhibited stemness characteristics with strong sphere-forming and tumorigenesis ability. iOVCAR-3-OSKM cells also showed malignant potential with higher drug resistance to chemodrug, Paclitaxel (PTX) and migration ability. iOVCAR-3-OSKM was maintainable and expandable on feeder-dependent culture condition, it also preserved ovarian lineage differentiation abilities, which could well differentiate into OC cells with CK-7 and CA125 expressions and develop into an organoid mimic poor prognostic OC histological feature.

**Conclusions:**

The establishment of iOVCAR-3-OSKM not only allows us to fill the gap in the information on induced CICs in OC but also provides a potential strategy to develop personalized CICs and organoid models for treating OC in the near future.

**Supplementary Information:**

The online version contains supplementary material available at 10.1186/s12885-022-10042-3.

## Background

Ovarian cancer (OC) is the most lethal gynecological cancer. Every year, more than a quarter of a million women are diagnosed with OC, and nearly 207,252 patients die from the disease globally [1]. OC is a heterogeneous disease comprising several histological types and subtypes. Most OC cases exhibit an epithelial phenotype (epithelial ovarian cancer [EOC]), and 75% of EOC cases are high-grade serous carcinoma (HGSOC) [2]. Unfortunately, HGSOC is the deadliest carcinoma and is often associated with peritoneal and omental metastasis and lymph node invasion upon initial diagnosis [3]. HGSOC causes up to 80% of the mortalities among OC patients and consequently represents the most conspicuous clinical challenge in gynecological oncology [4].

The standard management in advanced EOC is primary debulking surgery (PDS), followed by chemotherapy, which mainly includes a combination of taxane and platinum adjuvant chemotherapy. Neoadjuvant chemotherapy (NACT) and delayed interval debulking surgery (IDS) are alternative options based on the perspective and retrospective studies, which showed a similar prognosis compared to PDS as well as increased optimal debulking rate, improved quality of life, and decreased surgery-related complications [5]. Most OC patients respond well to initial treatment. However, approximately 75% of patients develop chemoresistance, leading to recurrence [6].

Several studies have shown that cancer-initiating cells (CICs) in tumors are responsible for cancer progression and tumor relapse [7]. Within various cancers, CICs display specific characteristics and behaviors, such as self-renewal, clonal tumor initiation capacity, elevated drug efflux pump activity and clonal long-term repopulation potential, which are conducive to metastatic potential and resistance to therapy [8, 9]. Therefore, targeting CICs is critical for advanced cancer treatment [10]. Nevertheless, current knowledge on CIC mechanisms remains limited due to the difficulties in CIC detection and identification.

Conventionally, CICs can be isolated by separating the side population (SP) based on dye-efflux activities [11]. CICs are believed to have high transporter activity to expel chemotherapeutic drugs, which is associated with treatment failure. Nevertheless, the inefficiency of SP isolation limits the accessibility of personalized CIC modeling. Due to the toxic effects on isolated cells, the fraction of potential SP cells is usually very small.

CICs share properties with stem cells and adult stem cells in terms of gene networks essential for self-renewal and pluripotency [12, 13]. Accumulating studies have demonstrated increased expression of the four Yamanaka factors *OCT3/4, SOX2, KLF4,* and *c-MYC* (collectively referred to as *OSKM*) in the CICs of various cancers, including glioma, lung cancer, prostate cancer, and bone sarcoma; and this upregulation is associated with a poor prognosis for cancer patients [14–16]. Therefore, an efficient and feasible method for propagating CICs with high expression levels of the four factors could advance cancer research and facilitate the development of personalized therapy targeting CICs. For this purpose, generating CICs from cancer cells using induced pluripotent stem cell (iPSC) technology is a viable strategy. In several cancer types, evidence indicates that cancer cells from clinical samples or cell lines can acquire CIC properties through stemness gene activation [12, 17]. They also display plasticity via the reversible transitioning between stem-cell and non-stem-cell states [18]. However, there have been few reports concerning induced CIC properties from OC cells [19]. One recent study reported that reprogrammed PEO4 cells through *OSKM* factors exhibited elevated CIC markers, and cells were more resistant to chemical drugs and formed more colonies than the parental cells. However, the crucial characteristic of malignancylike tumorigenicity, migration ability, and further gene profile analysis were not assessed in their study [19].

Herein, we generated an expandable induced ovarian cancer initiating cell (iOCIC) model by transducing *OSKM* using an integration-free SeV gene delivery system*,* into an HGSOC cell line, OVCAR-3. According to our results, stemness was successfully evoked in iOCICs, with significantly elevated expression of CIC-related genes and increase sphere-forming ability. The cells also displayed enhanced tumorigenicity, migratory activity, and drug resistance, thereby representing the characteristics associated with tumor recurrence and poor clinical outcomes in advanced tumors. In addition, in vitro and in vivo assays revealed that iOCICs maintain ovarian lineage-specific differentiation ability, as well as the capability of presenting OC pathological features. Our findings in this study provide a procedure to generating expandable iOCIC which may support the development of personalized treatments using patient-derived iOCICs. Furthermore, we successfully established organoid cultures from the iOCICs. Although conventional two-dimensional (2D) cell cultures allow the rapid expansion of cancer cells, the major drawback of this technique is the inability to faithfully reproduce the clinical cancer spectrum [20]. Organoids represent in vitro self-developing three-dimensional (3D) structures grown from stem cells, comprising organ-specific cell types that exhibit spatially-restricted lineage commitments, thereby reproducing key features of the tissue of origin [21, 22].

## Methods

### Medical records collection, immunohistochemistry (IHC) and statistics of stemness gene expression in clinical samples

Eighteen HGSOC patients receiving surgery in the National Taiwan University Hospital from January 2008 to December 2016 were consecutively enrolled in this cohort study. All patients were diagnosed by the validation of clinical properties, imaging examination and pathologic analysis. This investigation was approved by the Research Ethics Committee of the National Taiwan University Hospital (No. 200706002R and 202006036R), and all patients provided informed consent. The baseline demographic, pathological and clinical characteristics of all patients, including age, differentiation type, tumor size, and tumor-node-metastasis (TNM) stage, were collected. The TNM stage was assessed according to the 7^th^ edition of the American Joint Committee on Cancer (AJCC) cancer staging manual.

For immunohistochemical staining, tissues were fixed with formalin, and paraffin-embedded Sects. (5 μm thick) were stained with a Ventana BenchMark XT automated slide-staining system (Ventana Medical Systems, Inc., Roche, Tucson, Arizona, USA). The antibodies used in this study are listed in Additional file 1: Table S1. OCT4, SOX2, and NANOG expression in tumor tissues was quantified with a histological score (HSCORE) ranging from 0 (no staining) to 3 (maximal staining), which was calculated to assess IHC, and the computational formula was as follows: HSCORE = Σpi(i), where “pi” represents the percentage of positive cell counts in total cell counts, and “i” represents the intensity. An HSCORE of 0.05 was considered as the threshold which can distinguish high expression and low expression of the protein expression in IHC staining. Data are presented as the mean ± standard deviation (SD), and statistical analyses were carried out using SPSS 22.0 (IBM Corp., Armonk, NY, USA). Kaplan–Meier analysis was performed, and the log-rank test was used to investigate the correlation between OCT4, SOX2, and NANOG expression and overall survival (OS). A *p* value ≤ 0.05 was considered to indicate significance.

### Cell lines and cell culture

The human OC cell line OVCAR-3 was provided by Dr. Wen-Fang Cheng (Graduate Institute of Oncology, College of Medicine, National Taiwan University). Cells were cultured in Dulbecco's modified Eagle's medium (DMEM; Gibco, Thermo Fisher Scientific, Inc., Waltham, MA, USA) containing 10% fetal bovine serum (FBS), penicillin (100 Units/ml) and streptomycin (PS, 100 µg/ml) (All form Gibco); and the cultures were maintained in a humidified incubator at 37 °C with 5% CO_2_. The human embryonic stem cell (hESC) line H9 and the iPSC line generated from human normal peripheral blood mononuclear cells (iPBMCF) were maintained on mitomycin-c-treated mouse embryonic fibroblast cells (MEFs) with DMEM/F12 medium supplemented with 20% KnockOut Serum Replacement (KOSR), 10 ng/mL bFGF, 0.1 mM nonessential amino acids (NEAAs), 1 mM glutamine (all from Gibco), and 0.1 mM β-mercaptoethanol (Invitrogen, Thermo Fisher Scientific Inc., Waltham, MA, USA). Cultures were maintained in a humidified incubator at 37 °C with 5% CO_2_. The medium was changed daily; and the hESCs and hiPSCs were passaged via microdissection once a week (split ratio 1:3). In addition, Array Comparative Genomic Hybridization (aCGH) were performed for genome-wide screening to confirm the 45, XX karyotype of OVCAR-3 and iOVCAR-OSKM, and the consistent detectable genetic alteration in these two cells (Additional file 11: Figure S10).

### Generation of iOCICs utilizing the Sendai virus (SeV) reprogramming system

The CytoTune™-iPS 2.0 Sendai Reprogramming Kit was used for the generation of iOCICs via transduction of Yamanaka factors following the manufacturer's protocol (Invitrogen). The TRA-1–60 Alexa Fluor™ 488 Conjugate Live Imaging Kit (Invitrogen) was used to select Tra-1–60-positive colonies of mitomycin-c-treated MEFs. These cells were cultured in Primate ES cell medium (ESRC) (Reprocell, Beltsville, MD, USA) containing 10 ng/ml bFGF (Sigma–Aldrich, St. Louis, MO, USA). Cultures were maintained in a humidified incubator at 37 °C with 5% CO_2_; and the medium was changed daily. iOCICs were passaged via microdissection once every week or two weeks.

### RNA isolation and reverse-transcription polymerase chain reaction (RT–PCR)

Total RNA was extracted with TRIzol reagent (Invitrogen) and treated with DNaseI (Biorad) to eliminate the contamination of genomic DNA and isolated using the RNeasy Plus Mini Kit (QIAGEN, Hilden, Germany) following the manufacturer's instructions. A 1 µg aliquot of total RNA was reverse transcribed to single-strand cDNA by using the RevertAid H Minus First Strand cDNA Synthesis Kit (Thermo Fisher Scientific Inc., Waltham, MA, USA). Polymerase chain reaction (PCR) was performed using the ProFlex™ 96-well PCR system (Applied Biosystems, Thermo Fisher Scientific Inc., Waltham, MA, USA). Elimination of the exogenous reprogramming SeV vector was determined using SeV-specific primers. Endogenous gene expression, including pluripotent genes *(OCT3/4, SOX2, KLF4, c-MYC, and NANOG),* epithelial-mesenchymal transition (EMT)-related genes *(SNAIL, SLUG, TWIST, FIBRONECTIN, VIMENTIN, N-CADHERIN, and E-CADHERIN),* and a housekeeping gene, *GAPDH,* was amplified. The oligonucleotide primer sequences are listed in Additional file 1: Table S2. The thermal cycling conditions were as follows: initial denaturation at 95 °C for 3 min, followed by 35 cycles of amplification (95 °C for 30 s, 55–60 °C for 1 min, and 72 °C for 1 min) and a final extension at 72 °C for 10 min. PCR products were analyzed by 1.8% agarose gel electrophoresis.

### Quantitative reverse transcription polymerase chain reaction (qRT–PCR)

The mRNA expression level of OVCAR-3 and iOVCAR-3-OSKM was assessed. Total RNA was extracted with TRIzol reagent (Invitrogen) and treated with DNaseI (Biorad) to eliminate the contamination of genomic DNA and isolated using the RNeasy Plus Mini Kit (QIAGEN, Hilden, Germany) following the manufacturer's instructions. A 1 µg aliquot of total RNA was reverse transcribed to single-strand cDNA by using the RevertAid H. qRT–PCR was performed using FastStart Universal SYBR Green Master Mix (Applied Biosystems) and was analyzed with a StepOne Plus™ Real-time PCR system (Thermo Fisher Scientific). Each sample contained 50 ng cDNA in final volume 10ul. The thermal cycling conditions were as follows: initial denaturation at 95 °C for 3 min, followed by 35 cycles of amplification (95 °C for 3 s, 55 °C for 30 s, and 72 °C for 30 s) and a final extension at 72 °C for 10 min. The relative mRNA expression levels of endogenous pluripotent genes *(OCT3/4, SOX2, KLF4, c-MYC, and NANOG)*, previously reported OCIC markers (*CD133, CD117, CD24,* and *CD44)*, and chemoresistance-related genes (*ABCG2* and *ALDH1*) were assessed using the oligonucleotide primers listed in Additional file 1: Table S2.

### Immunofluorescence (IF) staining

Briefly, the cells were fixed with 4% paraformaldehyde (Sigma–Aldrich), permeabilized with 0.2% Triton-100 (Thermo Fisher Scientific), blocked with 3% BSA (Sigma–Aldrich) and incubated with primary antibodies against pluripotent proteins (OCT3/4, TRA-1–60, NANOG, and CD133) and OC lineage differentiation-related proteins (CK7 and CA125) at 4 °C overnight. Cells were then incubated with appropriate secondary antibodies (1:250 diluted in Dulbecco’s phosphate-buffered saline [DPBS], Corning, Corning, New York, USA) at room temperature for 1 h. The antibodies used in this study are listed in Additional file 1: Table S1. Nuclei were stained with Hoechst 33,342 (Thermo Fisher Scientific) stain solution (diluted in DPBS, Corning, Corning, NY, USA) for 5 min. Fluorescence images were captured with an inverted fluorescence microscope (ECLIPSE TE2000-U, Nikon, Tokyo, Japan).

### Flow cytometry (FCM)

Cells were trypsinized into single cells and fixed and permeabilized using the BD Cytofix/Cytoperm Kit (BD Pharmingen™, San Jose, CA, USA). A single-cell suspension of cultured cells was immunostained with primary antibodies against pluripotent proteins (OCT3/4, TRA1-60, and NANOG), previously reported OCIC markers (CD133, CD117, CD24, and CD44), chemoresistance-related proteins (ABCG2 and ALDH1), OC lineage differentiation-related markers (CK7 and CA125), and appropriate secondary antibodies. After washing, the cells were resuspended in DPBS (Corning) containing 2% FBS (Gibco). The samples were analyzed with a FACS Aria II instrument (BD Pharmingen™). The percentages of OCT3/4-, TRA1-60-, NANOG-, CD133-, CD117-, CD24-, CD44-, ABCG2-, ALDH1-, CK7-, and CA125-positive cells were recorded. The antibodies used in this study are listed in Additional file 1: Table S1.

### In vitro differentiation

A differentiation procedure was used to determine the in vitro differentiation of iOVCAR-3-OSKM cells. Briefly, iOVCAR-3-OSKM cells were detached with Accutase solution (Gibco) and seeded onto 0.4% pluronic acid precoated ultralow attachment 6-well plates (Corning) with the treatment of 10 μM of Rock inhibitor (Y27632) (Sigma–Aldrich) and cultured with ESRC (Reprocell, Beltsville) containing 10 ng/ml bFGF (Sigma–Aldrich) for 7 days. After 7 days, the cells were collected and transferred onto 0.1% gelatin-coated plates and cultured in DMEM with 10% FBS (Gibco) for 14 days. The cells were fixed on day 14 with 4% paraformaldehyde (Sigma–Aldrich). Antibodies specific for the OC lineage markers CK-7 and CA125 were used for IF. Appropriate secondary antibodies were used for detection. The antibodies used in this study are listed in Additional file 1: Table S2.

### Sphere-formation assay

A total of 10^5^ cells were transferred to ultralow attachment multiwell plates (Corning) in ESRC (Reprocell, Beltsville, MD, USA) containing 10 ng/ml bFGF, 10 mg/ml human insulin, 100 mg/ml BSA (all from Sigma–Aldrich) and 100 mg/ml human transferrin (Roche, Basel, Switzerland); and incubated as described above for 14 days. The numbers of spheres were manually counted using an inverted microscope (ECLIPSE TE2000-U, Nikon, Tokyo, Japan).

### Tumorigenicity assay

For animal experiments, to decide an appropriate cell dose which could identify the tumorigenicity of OVCAR-3 and iOVCAR-3-OSKM, ten 6-week-old female immunodeficient NOD.CB17-Prkdcscid/NcrCrl mice (LASCO, Taiwan) were randomized into two groups with five mice each (Group 1: injected with OVCAR-3 and Group 2: injected with iOVCAR-3-OSKM).We used sequential tenfold dilutions of cells; a total of 10^7^, 10^6^, 10^5^, 10^4^, and 10^3^ OVCAR-3 or iOVCAR3-OSKM cells in 100 µl serum-free DPBS (Corning) were subcutaneously injected into both dorsal flanks of two groups of NOD-SCID mice (LASCO, Taiwan). The mice underwent continuous observation after subcutaneous inoculation. Four months after tumor cell injection, all mice were anesthetized with isoflurane before sacrifice, and subcutaneous tumors were surgically excised. Tumor incidence was counted and tumor volume was measured and calculated as volume = 0.5 × L × W^2^ (L: length, W: width). Each animal served as an experimental unit and none of the data collected was excluded.

To further clarify the tumorigenicity between OVCAR-3 and iOVCAR-3-OSKM. We performed a repeat animal experiment. Sixteen 6-week-old female immunodeficient NOD.CB17-Prkdcscid/NcrCrl mice (LASCO, Taiwan) were randomized into two groups with eight mice each (Group 1: injected with OVCAR-3 and Group 2: injected with iOVCAR-3-OSKM). A total 10^3^ OVCAR-3 or iOVCAR-3-OSKM cells were subcutaneously injected into both dorsal flanks of two groups of NOD-SCID mice. Four months after tumor cell injection, all mice were anesthetized with isoflurane before sacrifice, and subcutaneous tumors were surgically excised. Tumor incidence was counted and tumor volume was measured and calculated as volume = 0.5 × L × W^2^ (L: length, W: width). Each animal served as an experimental unit and none of the data collected was excluded.

Researchers were not blinded to both groups, as monitoring tumor mass was part of humane animal study endpoints. Mice in this study were housed 4–5/cage and acclimated to the temperature-controlled (22 ± 1 °C) vivarium with a 14:10 light:dark cycle. Rodent chow (Teklad 7912) and water were available ad libitum throughout the study and cotton nestlets and plastic huts were provided for nesting. The experiments were reviewed and approved by the Animal Ethics and Research Committee of National Taiwan University (No. 2016206) and conducted following institutional guidelines.

### MTT chemoresistance assay

The cell viabilities of iOVCAR-3-OSKM cells and parental cancer cells after paclitaxel (PTX, Sigma–Aldrich) exposure was measured by MTT colorimetric assays (Roche, Basel, Switzerland). DMSO was used to dissolve paclitaxel. A total of 10^4^ cells were seeded in 96-well plates and cultured for 24 h. Then, the medium was replaced with DMEM containing 0.01–10 µM PTX, and the absorbance at 595 nm was measured using a microtiter plate reader after 72 h-incubation. Cell viability was calculated as the ratio of absorbance values for the treated sample versus the same sample incubated in DMEM without PTX treatment.

### Hematoxylin–eosin (H&E) staining and IHC

Mice were sacrificed for histological analyses 10 weeks after subcutaneous transplantation with cells. Tumors were surgically excised and fixed with 4% formalin, followed by paraffin embedding. Then, the tissue specimens were obtained for H&E and IHC staining with anti-human cytokeratin 7 (CK7) rabbit monoclonal antibody or anti-human cancer antigen 125 (CA125) mouse monoclonal antibody using the avidin–biotin immunoperoxidase method. The antibodies used in this study are listed in Additional file 1: Table S1.

### Dye efflux activity analysis

iOVCAR-3-OSKM cells were harvested in ESRC (Reprocell, Beltsville, MD, USA) containing 2% FBS (Gibco) and 1 mM HEPES (Sigma–Aldrich), followed by staining with 2.5 µg/ml Hoechst 33,342 (Thermo Fisher Scientific) with or without coadministration of verapamil (VM) (Sigma–Aldrich) at 0, 50, or 250 µM for 90 min at 37 °C. Tubes were gently inverted every 30 min. After incubation, the cells were washed and resuspended in DPBS containing 2% FBS and 1 mM HEPES. The cells were then counterstained with 2 µg/ml PI (Sigma–Aldrich) to label dead cells, passed through a 35 µm mesh filter, and kept on ice for FCM and sorting. The cells were analyzed and sorted with a FACSAria™ II (BD Bioscience). Hoechst dye was excited with a UV laser (355 nm), and the fluorescence was measured with 670/50 (Hoechst Red) and 450/50 (Hoechst Blue) filters.

### Trans-well migration assay

Corning® Transwell® CLS3422 6.5 mm Transwell with 8.0 μm pore polycarbonate membrane inserts (Merck, Sigma–Aldrich, St. Louis, MO, USA) was used for Transwell assays. A total of 10^5^ cells were seeded into the upper layer of cell culture inserts with 100 µl serum-free medium, and medium containing 10% FBS was placed below the permeable membrane. After 24 h, cells that migrated through the membrane were stained and counted.

### Organoid culture

Briefly, 3 × 10^5^ single cells were suspended in medium containing 2% Matrigel (Corning) and 50 nM 17β‐estradiol (E2) (Merck, Sigma–Aldrich) and were seeded into culture dishes coated with 100% Matrigel, followed by incubation at 37 °C in 5% CO_2_ for 14 or 28 days. The number (> 20 µm organoids under 100X magnification) and diameter (10 organoids under 200X magnification) of organoids were calculated and statistically analyzed by ImageJ bundled with Java 1.8.0_172 software.

### Next-generation sequencing (NGS) analysis

More than 5 × 10^6^ of OVCAR-3, OVCAR-3 SP, and iOVCAR-3-OSKM cells were collected and used for RNA extraction with the QIAGEN RNA Isolation Kit. Purified RNA was quantified at OD260 nm using an ND-1000 spectrophotometer (Nanodrop Technology, USA), quantified through a Bioanalyzer 2100 instrument (Agilent Technology, USA) and the RNA 6000 LabChip Kit (Agilent Technology, USA). All RNA sample preparation procedures were carried out according to Illumina's official protocol. The SureSelect XT HS2 mRNA Library Preparation Kit (Agilent) was used for library construction followed by AMPure XP bead (Beckman Coulter, USA) size selection. The sequence was determined using Illumina's sequencing-by-synthesis (SBS) technology (Illumina, USA). Sequencing data (FASTQ reads) were generated using Welgene Biotech's pipeline based on Illumina's base calling program bcl2fastq v2.20. Briefly, the program was used to convert BCL files from all Illumina sequencing platforms into FASTQ reads. Adaptors are short nucleotide sequences that have to be ligated to every single DNA molecule during library preparation and allow DNA fragments to bind to a flow cell for next-generation sequencing. Removal of adaptor sequences, a process called 'adaptor trimming', or clipping, is one of the first steps in analyzing NGS data. Quality trimming was performed to remove low-quality reads/bases. Lower quality bases from the 3' end were removed using a sliding-window approach as the per base quality gradually dropped toward the 3' end of reads. Both adaptor clipping and sequence quality trimming were performed using Trimmomatic v0.36 with a sliding-window approach. Differential expression analysis was performed using StringTie (StringTir v2.1.4) and DEseq (DEseq v1.39.0) or DEseq2 (DEseq2 v1.28.1) with genome bias detection/correction and Welgene Biotech's in-house pipeline. Functional enrichment of the differentially expressed genes (DEGs) in each experiment was performed using clusterProfiler v3.6. Genes with low expression levels (TPM value < 0.3) in either or both the treated sample and the control sample were excluded. Genes with |log2 FC|> 2 were defined as DEGs. The intersecting DEGs of iOVCAR-3-OSKM/OVCAR-3 and SP/OVCAR-3 with > 5 or < 0.2 were selected and assessed with Gene Ontology (GO) analysis. The heatmap was created by Python 3.10 with seaborn0.11.2.

### Statistical analysis

The results were analyzed and plotted with GraphPad Prism™ software (version 7.0a). All values are expressed as the mean ± SD. Student’s t test was used to compare the means between two groups. A *P* value (*P*) < 0.05 was considered to denote statistical significance.

## Results

### High expression of pluripotent genes was correlated with worse clinical outcome in high-grade serous ovarian carcinoma (HGSOC) patients

**Fig. 1:**
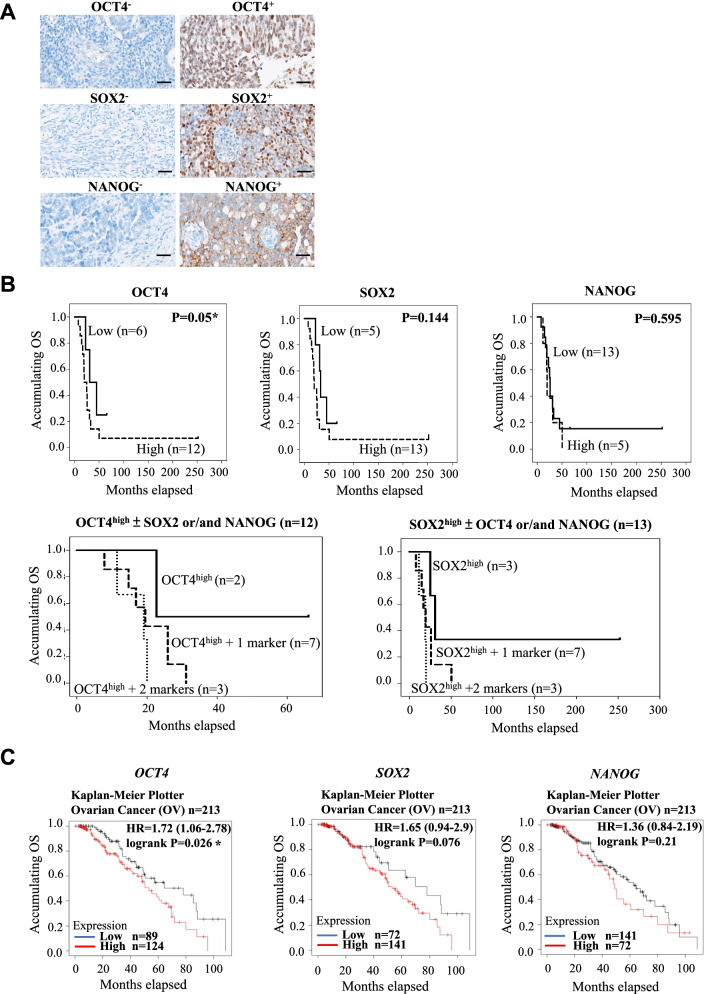
Expression of pluripotent genes and the correlation with OS in OC. **A** Immunohistochemical (IHC) staining of OCT4, SOX2, and NANOG expression in clinical tumors. OCT4-, SOX2-, and NANOG- indicate no primary antibody incubation during the staining procedure (original magnification, × 400). **B** Correlation of each single marker and OCT4^high^ or SOX2^high^ plus other markers with OS in operative patients with OC. Statistical analysis was carried out using SPSS 22.0 (IBM Corp., Armonk, NY, USA). Kaplan–Meier curves were used to evaluate the correlation of highly expressed markers with OS. Comparisons of two groups were made by the log-rank test. *P < 0.05 was considered significant. **(C)** Correlation of each marker with OS in 213 patients with grade 2 + 3 serous ovarian carcinoma containing Paclitaxel treatment from the dataset of Kaplan–Meier Plotter (https://kmplot.com/analysis/). Kaplan–Meier curves were used to evaluate the correlation of highly expressed markers with OS. Comparisons of two groups were made by the log-rank test. *P < 0.05 was considered significant

To investigate the association of CIC marker (OCT4, SOX2, and NANOG) expression with overall survival (OS) in patients, tissues from 18 HGSOC patients who had undergone surgical resection were used to perform immunohistochemical (IHC) staining. The characteristics of detected patients were shown in Table 1; and the negative and positive staining of OCT4, SOX2, and NANOG are shown in Fig. 1A. The HSCORE ranged from 0 (no staining) to 3 (maximal staining) was calculated as follows based on the IHC results: HSCORE = ΣPi(i), where “pi” represents the percentage of positive cells among total cells, and “i” represents the intensity. An HSCORE of 0.05 was considered as threshold for distinguishing the high and low expressions of detecting markers. As shown in Table 2, there were 12 (67%) patients with high OCT4 expression and 6 (33%) patients with low OCT4 expression. Thirteen (72%) patients had high SOX2 expression, while 5 (28%) patients had low SOX2 expression. For NANOG, 5 (28%) patients had high NANOG expression, while 13 (72%) patients had low NANOG expression. The co-expressing status of OCT4, SOX2, and NANOG in tumor tissues was also evaluated; and there were 2 (11%) patients with no marker expressed, 5 (28%) patients with only one marker expressed, and 8 (44%) patients with two markers significantly expressed [the expression groups included 1) the OCT4^high^ and SOX2^high^ group (*n* = 6, 33%); 2) the OCT4^high^ and NANOG^high^ group (*n* = 1, 6%); and 3) the SOX2^high^ and NANOG^high^ group (*n* = 1, 6%)]. In addition, three (17%) patients expressed all three markers at high levels (Table 3).

**Table 1:** Baseline characteristics of HGSOC patients

Parameters	OC patients n (%), total patients No. 18
Age (yrs)	53.72 ± 10.73
Tumor size (cm)	
< 5	6 (33)
≥ 5	12 (67)
Differentiation type	
Well differentiated	0 (0)
Moderately differentiated	2 (11)
Poorly differentiated	16 (89)
T stage	
T3	18 (100)
N stage	
N0	1 (6)
N1	17 (94)
M stage	
M0	15 (83)
M1	3 (17)
TNM stage	
III	15 (83)
IV	3 (17)

*OC* Ovarian cancer

Dara is presented as mean ± SD or counts (with or without percentage)

**Table 2:** OCT4, SOX2 and NANOG expression in OC tumor tissues

Parameters (*n* = 18)	OCT4^high^	OCT4^low^	SOX2^high^	SOX2^low^	NANOG^high^	NANOG^low^
Tumor tissue (%)	12 (67)	6 (33)	13 (72)	5 (28)	5 (28)	13 (72)

Data are presented as counts (percentage). The histological score (HSCORE) ranged from 0 to 3 and was calculated to assess immunohistochemical staining by the following formula: HSCORE = ΣPi(i) where “pi” represents the percentage of positive cell counts in total cell counts, and “i” represents the intensity). An HSCORE of 0.05 was considered as a threshold to distinguish high expression and low expression

**Table 3:** Co-expression of OCT4, SOX2 and NANOG in OC tumor tissues

Parameters	OC patients (*n* = 18)
Patients with no highly expressed marker (%)	2 (11)
Patients with only one highly expressed marker (%)	5 (28)
Patients with two highly expressed markers (%)	8 (44)
OCT4^high^ and SOX2^high^	6 (33)
OCT4^high^ and NANOG^high^	1 (6)
SOX2^high^ and NANOG^high^	1 (6)
Patients with three highly expressed markers (%)	3 (17)

Data are presented as counts (percentage)

* OC* Ovarian cancer

Moreover, the correlations between OCT4, SOX2, and NANOG expression with OS were also assessed. The mean time of OS was 38.9 months, and the 5-year survival rate was 11.1%. Kaplan–Meier analysis indicated that HGSOC patients with OCT4^high^ status displayed a trend with worse OS than those with OCT4^low^ status (*p* = 0.0503) (Fig. 1B), while no association of SOX2 (*p* = 0.144) (Fig. 1B) or NANOG expression (*p* = 0.595) (Fig. 1B) with OS was observed. To further evaluate the contribution of these factors to poor OS, we analyzed the correlations of high expression of OCT4 and SOX2 plus other markers with OS. The NANOG^high^ group was not included into this analysis owing to the limited case numbers that no sample expressed NANOG^high^ only and only two cases showed high expression of NANOG plus another marker in our dataset. As expected, patients with OCT4 or SOX2 high expression combined with highly expression of another marker may present worse OS than those only with high expression of OCT4 or SOX2 alone (Fig. 1B); and the patients expressed high levels of all the three markers showed the worst OS. Moreover, the correlation of the numbers of highly expressed markers with OS was also assessed. The data indicated that patients with at least two highly expressed markers were have worse OS compared with those with one or no highly expressed marker expression (*p* < 0.025, Additional file 2: Figure S1); while all three highly expressed markers were correlated with worse OS than two or fewer positive markers (*p* < 0.018) (Additional file 2: Figure S1).

For further validating our findings in clinic, we also choose an ovarian cancer tissue array as an independent cohort to perform IHC stating. The array contained 18 EOC patients including 8 (44%) HGSOC, 2 (11%) endometrioid adenocarcinoma, 7 (39%) adenocarcinoma and 1 (6%) mucinous adenocarcinoma cases; and the characteristics of this cohort were shown in Additional file 1: Supplementary Table 5. The number of single or co-expressing status of OCT4, SOX2, and NANOG in tumor tissues was calculated in Additional file 1: Supplementary Table S6 and Table S7. Kaplan–Meier analysis indicated that patients with OCT4^high^ status had worse OS than those with OCT4^low^ expressions (*p* = 0.012), while no association was found in SOX2 (*p* = 0.062) or NANOG expression (*p* = 0.145) (Additional file 13: Figure S12 A). As expected, patients having much more markers highly expressed were have much worse OS compared with those with no or less highly expressed marker expression (*p* = 0.035 for at least one vs. no, 0.001 for at least two vs. one or no, 0.024 for all three vs. two or less, Additional file 13: Figure S12 B). Although there were only 8 HGSOC cases enrolled in this cohort, similar trend could be also found that when much more pluripotent makers highly expressed in the tumor tissues, the HGSOC patients might have much worse clinical outcome compared with those with no or less markers expressed (*p* = 0.007 for OCT4, 0.054 for SOX2, 0.053 for NANOG, and 0.007 for at least two vs. less, Additional file 14: Figure S13).

In addition to IHC staining, we also used two public database, Kaplan–Meier Plotter (https://kmplot.com/analysis/) [23] and UCSC Xena (http://xena.ucsc.edu) [24], to re-examine the correlations between the markers and OS in ovarian cancer patients. The database of Kaplan–Meier Plotter includes the mRNA profiling from 213 patients with grade 2 + 3 serous ovarian carcinoma with paclitaxel treatment. The results indicated that patients with *OCT4*^high^ expressions display worse OS compared with those of *OCT4*^low^ expressions (*p* = 0.026), while none significant association was observed in *SOX2* (*p* = 0.076) or *NANOG* (*p* = 0.21) expressions (Fig. 1C). However, the HR of these three genes suggested all of them are risk factors for OS in ovarian cancer patients (the HR = 1.72 for *OCT4*, 1.65 for *SOX2* and 1.36 for *NANOG*. On the other hand, we also analyzed the associations using the RNAseq data of 376 patients with primary ovarian cancer from GDC TCGA Ovarian Cancer (OV) cohort in the database of UCSC Xena. As expected, patients with high *OCT4* expressions displayed worse OS compared with those of low *OCT4* expressions, but the result still not reached significant (*p* = 0.08868). And similar findings of none significant association were also observed in *SOX2* (*p* = 0.5728) and *NANOG* (*p* = 0.1316) analyses (Additional file 10: Figure S9A). In addition, we also evaluated these gene expression levels in different histologic grades. The results showed that the *OCT4* expression level was significant elevated in high grade patients (*p* = 0.01357) (Additional file 10: Figure S9B). All these results from public domain were consistent with what we observed in our study cohort.

### Establishment of an iOCIC model by transduction of OCT3/4, SOX2, KLF4, and c-MYC

Based on our clinical data, that high expression of OCT4 were correlated with poor OS in patients; the combination of more pluripotent marker expression seems synergistically correlated with worse OS. Targeting CICs could become a new choice in clinics that may significantly improve the clinical outcome of OC. This let establishing an in vitro culture system of CICs be benefit for the development of CIC targeting therapy.

**Fig. 2:**
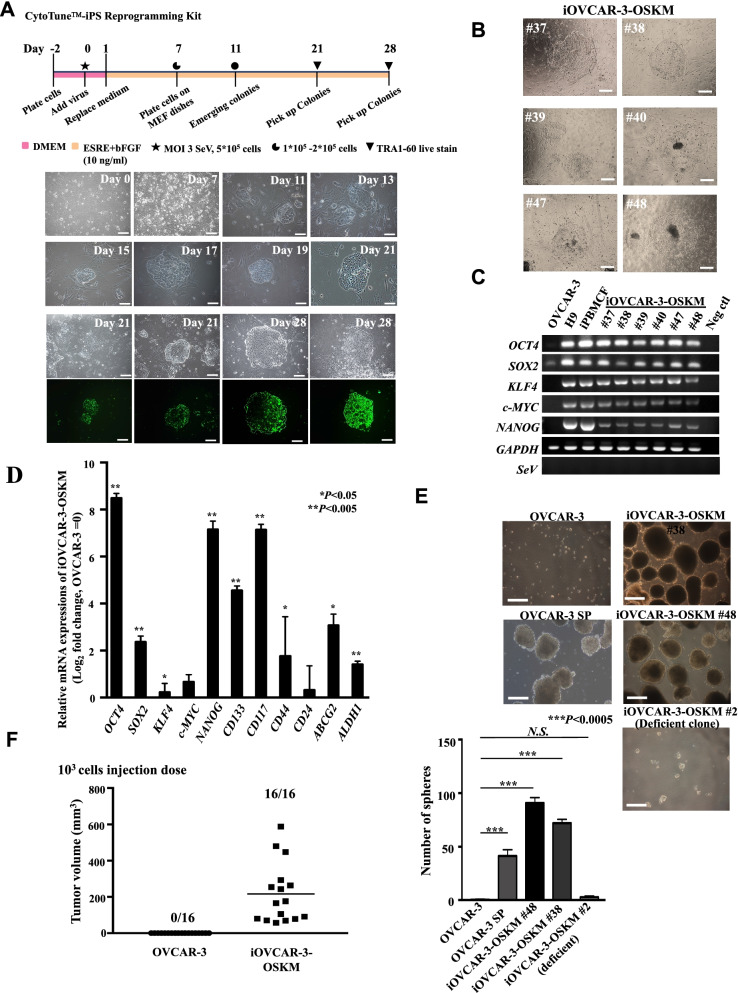
Establishment of the iOVCAR-3-OSKM model, which exhibited stemness and cancer-initiating cell properties. **A** Schematic of the transduction procedure and establishment of iOCICs. The cells were infected with SeV containing four factors (*OSKM*) at day 0 and replated onto MEF dishes. Tra-1–60 positive colonies were picked up on day 21 and day 28. Scale bars: 200 µm. **B** Morphology of iOVCAR-3-OSKM colonies. Six-day 7-iOVCAR-3-OSKM clones on feeder cells. Scale bars: 200 µm. **C** Pluripotent gene expression and SeV silencing in iOVCAR-3-OSKM clones. Expression of endogenous pluripotent genes (*OCT4, SOX2, KLF4, and NANOG*), *SeV*, and the housekeeping gene *GAPDH*. cDNA of human ESCs (H9 cell line) and a human induced pluripotent cell line (iPBMCF) were used as positive controls. Neg Ctl: negative control (PCR mixture without cDNA). The samples derive from the same experiment and gels were processed in parallel. **D** qRT–PCR of the gene expression of four factors and markers previously reported to be related to OCICs in iOVCAR-3-OSKM cells. mRNA expression levels were normalized to those of *GAPDH.* Relative expression levels compared to those in parental OVCAR-3 cells are shown in log2 scale (*n* = 3). Error bars indicate SEM. *P < 0.05, **P < 0.005, Student's t test. **E** Sphere-formation ability in vitro*.* A total of 10^5^ cells were plated on low attachment dishes and cultured for 14 days. The numbers of spheroids (≥ 25 µm) were counted under a microscope. Spheres numbers of iOVCAR-3-OSKM #48, #38 and SP were significantly increased compared #2 and parental OVCAR-3 cells. The error bars indicate the SD. ***P < 0.0005, Student’s t test. *NS* means nonsignificant. Scale bars: 200 µm. *NS* means nonsignificant. **F** Tumorigenicity of iOVCAR-3-OSKM cells after implantation in the subcutaneous regions of immunodeficient NOD-SCID mice. A total of 10^3^ cells were subcutaneously injected into both flanks of immunodeficient female NOD-SCID mice (*n* = 8). Tumor volume and tumor formation rate (*n* = 16) are shown

We used an integration-free SeV gene delivery system to transduce *OCT3/4, SOX2, KLF4,* and *c-MYC* (hereafter, *OSKM)* into a human OC cell line, OVCAR-3 which was isolated from HGSOC. The schematic of the transduction procedure is shown in Fig. 2A. Briefly, we performed transfection on day 0 and replaced the culture medium to remove the virus after 24 h. On day 7, transduced cells were replated onto feeder cells with different cell numbers (1 × 10^5^ and 2 × 10^5^ cells). On day 11, we observed colonies (Fig. 2A). Notably, the colony numbers in the 1 × 10^5^ group were greater than those in the 2 × 10^5^ group at day 14. Transfection efficiency was calculated and is listed in Additional file 1: Table S3. The number of colonies undergoing early reprogramming was > 300 in the 1 × 10^5^ group and < 100 in the 2 × 10^5^ group. The pluripotent gene Tra1-60 was used as a selection marker. Tra1-60-positive colonies were selected on day 21 or day 28 followed by single colony subcloning (Fig. 2A); and the single colonies were maintained on MEF feeder cells with weekly or biweekly subculture. Notably, conspicuous morphological changes in the colonies were attributable to the transduced genes (Fig. 2B). RT–PCR was performed to confirm the mRNA expressions of pluripotent genes and the silencing of SeV after 10 passages. The data showed that the *OSKM* genes were endogenously expressed, and exogenous *SeV* expression was completely eliminated (Fig. 2C). Transduced cells that endogenously expressed *OSKM* genes were termed iOVCAR-3-OSKM cells. The expressions of stemness-related proteins, Oct-4, TRA-1–60, NANOG, and CD133, were also assessed by IF staining (Additional file 3: Figure S2). Herein, inducible and maintainable expression of multiple pluripotent genes was achieved by reprogramming.

### iOVCAR-3-OSKM cells exhibited stemness characteristics

In order to scrutinize the initiating cell status and identity of iOVCAR-3-OSKM cells, we evaluated the expression levels of several ovarian cancer initiating cell (OCIC) markers that previously reported like *CD133* [25], *CD177* [26], *CD24* [27], *CD44* [28], *ABCG2* [29], and *ALDH1* [30] in cells. The results showed that the mRNA expression levels of almost all target genes significantly increased in iOVCAR-3-OSKM cells compared with the parental OVCAR-3 cells (Fig. 2D, P < 0.05, n = 3). Flow cytometry analysis also indicated that increasing expression levels of the pluripotent genes OCT4, TRA1-60, NANOG and the OCIC cell markers CD117, CD133, CD44, and ABCG2 were observed in iOVCAR-3-OSKM cells (Additional file 4: Figure S3, *P* < 0.05, *n* = 3). All these markers contribute to the regulation of self-renewal, differentiation, and chemoresistance and indicate that iOVCAR-3-OSKM cells may expressed like a CICs.

For confirming the stemness of iOVCAR-3-OSKM cell, another typical characteristic of CICs, sphere-forming ability, was evaluated [31]. For this examination, a total of 10^5^ cells were transferred to ultralow attachment multicell plates and cultured for 14 days. In addition to the two well-established clones (iOVCAR-3-OSKM #38 and iOVCAR-3-OSKM #48), the side population (SP) cells and the parental OVCAR-3 cells were used as the positive and negative controls; and the number of spheroids in each group was calculated. As Fig. 2E shown, iOVCAR-3-OSKM #38 and #48 cells clearly demonstrated the ability to form spheres. In contrast, spheroids were difficult to find in the parental OVCAR-3 and OVCAR-3 #2, one clone with unsuccessful transduction, groups.

To further assess the phenotypic traits of iOVCAR-3-OSKM cells, the ability of cell growth was evaluated by in vitro cell proliferation assays. The data showed that the number of iOVCAR-3-OSKM cells was significantly lower than the number of parental OVCAR-3 cells at 72 h after seeding with 2 × 10^5^ cells (Additional file 5: Figure S4, *P* < 0.05, *n* = 3), suggesting that *OSKM* transduction essentially transforms cells into a relatively quiescent state, which may more resistant to anti-cancer therapeutics.

Cancer cells endowed with stem cell properties are maintained in a quiescent slow-growing state that protects them from the effects of antiproliferative anticancer drugs [32]. CICs are capable of supporting tumor regrowth, which leads to a more aggressive disease in vivo [33]. To examine the in vitro tumorigenicity of iOVCAR-3-OSKM cells, we first subcutaneously transplanted 10^7^, 10^6^, 10^5^, 10^4^, and 10^3^ OVCAR-3 cells or iOVCAR-3-OSKM cells into female immunodeficient NOD-SCID mice, and the tumor formation rate and tumor volume were recorded (Additional file 1: Table S4). The data showed that 100% engraftment was observed at all mice injected with iOVCAR-3-OSKM xenografts. In contrast, parental OVCAR-3 cells displayed lower tumorigenicity, which indicated that they were incapable of generating tumors at injection doses of 10^4^ and 10^3^ cells (Additional file 1: Table S4). To further clarify the difference in tumorigenicity between iOVCAR-3-OSKM and parental cancer cells, we repeated the tumorigenesis experiment using 10^3^ cells (n = 16); in our result, the tumor formation rate and tumor volume were significantly higher in the group of iOVCAR-3-OSKM cells compared with the group of parental cells (Fig. 2F). All the data support iOVCAR-3-OSKM cells as a promising CIC model that exhibits the majority of the previously reported criteria for characterizing CIC signatures.

### iOVCAR-3-OSKM cells presented a gene profile that similar to the SP of OVCAR-3 cells

**Fig. 3:**
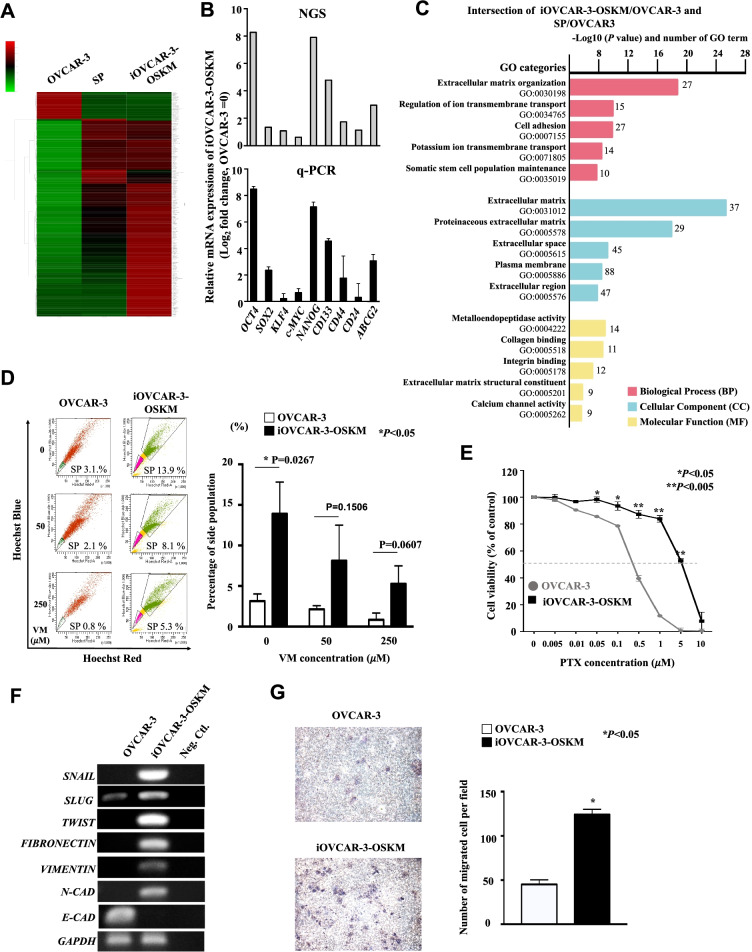
iOVCAR-3-OSKM showed SP enrichment and malignant properties. **A** The heatmap of DEGs. The row displays genes, and the column represents samples. Downregulated genes are displayed in green, and genes upregulated are displayed in red. The brightness of each color corresponds to the magnitude of the difference compared with the average value. **B** Validation of NGS data with q-PCR data by log2 gene expression fold changes. Error bars indicate SEM. **(C)** GO enrichment analyses of the intersecting DEGs of the iOVCAR-3-OSKM/OVCAR-3 and SP/OVCAR-3 datasets. Count represents the number of DEGs enriched in each term. The black trend line represents the log10 (*P* value). **D** Efflux activity of cells. The SP cells unlabeled by Hoechst 33,342 without VM and with 50 µM and 250 µM VM were counted. The left panel shows representative dot plots of labeled and unlabeled cells. The right panel shows statistics for SPs in each group (*n* = 3). Error bars indicate SD. *P < 0.05, Student’s t test. **E** Sensitivity to chemotherapeutic agents. The viability of the cells without PTX treatment was assumed to be 100%, and the viability of cells with PTX treatment was normalized to this value (*n* = 3). Error bars indicate SD. *P < 0.05, **P < 0.005, Student’s t test. **F** The expression of EMT-related genes. The endogenous EMT-related genes and the housekeeping gene *GAPDH* were amplified and separated by electrophoresis. Neg Ctl: negative control (PCR mixture without cDNA). The samples derive from the same experiment and gels were processed in parallel. **G** Migration ability of iOVCAR-3-OSKM cells. A total of 1 × 10^5^ cells were seeded into the upper layer of a cell culture insert with serum-free medium, and medium containing 10% FBS was placed below the permeable membrane for 24 h of incubation. The left panel shows images of migrating cells. The right panel provides statistics for migrating cell numbers (*n* = 3). Error bars indicate the SD. *P < 0.05, Student’s t test

In order to evaluate whether the OSKM transduced iOVCAR-3-OSKM cells are similar to the SP cells that isolated from OVCAR-3 cells, we performed RNA-seq and analyzed both of their gene expression profiles. Differentially expressed genes (DEGs) were identified using thresholds of |log2 FC|> 1 and a p value < 0.05. We totally obtained 2236 DEGs of iOVCAR-3-OSKM/OVCAR-3 cells and 1033 DEGs of SP/OVCAR-3 cells. For further evaluation, we chose the intersection of the two DEG groups and filtered them with strict thresholds of > 5 or < 0.2 and *p* value < 0.05. Finally, 258 intersecting DEGs were selected; the heatmap showed that the expression profile of intersecting DEGs in OVCAR-3-OSKM was similar to that of SP cells rather than OVCAR-3 cells (Fig. 3A). Further, the data were validated by q-PCR and the fold changes of gene expression were also be calculated (Fig. 3B). Moreover, we also performed GO analysis and found that several pathways including extracellular matrix organization (GO:0,030,198), regulation of ion transmembrane transport (GO:0,034,765), extracellular matrix (GO:0,031,012), proteinaceous extracellular matrix (GO:0,005,578), and metalloendopeptidase activity (GO:0,004,222) were enriched in our DEGs (Fig. 3C).

Next, we gated and quantified SP cells with or without coadministration of Verapamil (VM), which is a transporter inhibitor that can help us examine the dye-efflux activities of the cells. The results showed that the SP of parental OVCAR-3 cells was 3.1% of the cell population without VM cotreatment, and this number decreased to 2.1% and 0.8% with 50 µM and 250 µM VM cotreatment, respectively. SP was significantly decreased in parental OVCAR-3 cells with increasing doses of VM (Fig. 3D). In the iOVCAR-3-OSKM group, the number of SP cells was also slightly decreased in a dose-dependent manner with VM but was significantly increased compared with that in parental OVCAR-3 cells. Remarkably, the SP cells of the iOVCAR-3-OSKM group accounted for 13.9% of the cell population without VM cotreatment, 8.1% of the cell population in the presence of 50 µM VM cotreatment, and 5.3% of the cell population in the presence of high-dose (250 µM) VM cotreatment (Fig. 3D). All these results were consistent with the finding of the gene expression profiling and suggest significant SP enrichment in iOVCAR-3-OSKM cells.

### iOVCAR-3-OSKM cells showed malignant potential with higher drug resistance and migration ability than parental cells

Elevated drug efflux pump activity is one process employed by CICs to evade chemotherapy. To further examine the anticancer drug sensitivity of iOVCAR-3-OSKM cells, MTT assays were used to determine the survival rates of cells after treatment with paclitaxel (PTX), a first-line anti-OC drug. The data showed that the half-maximal inhibitory concentration (IC50) values of OVCAR-3 and iOVCAR-3-OSKM cells were 0.39 µM and 5.30 µM, respectively, indicating that iOVCAR-3-OSKM cells are more than 10 times less sensitive to PTX (Fig. 3E, P < 0.05, *n* = 3). iOVCAR-3-OSKM cells harbor an enriched SP with greater drug efflux activity. Such cells demonstrate chemoresistance to PTX, which is consistent with the increased expression of previously reported OCIC markers in iOVCAR-3-OSKM cells, such as CD133, CD117, and CD44, which are known to be associated with multiple drug resistance.

Additionally, EMT is also a crucial malignancy-related characteristic and a major driver of drug resistance. Several in vitro and in vivo studies have shown that cancer cells resistant to chemotherapeutic drugs acquire a mesenchymal phenotype [34]. Co-expression of stemness- and EMT-related genes has also been reported. Investigation of advanced-stage ovarian tumor sections, including metastatic cells isolated from ascites, revealed combined expression of EMT and CIC markers after chemotherapy. OC cells with stem cell properties and an EMT phenotype seem inclined to develop resistance to therapy [35]. Thus, we analyzed an epithelial marker, *E-CADHERIN*, and mesenchymal markers, *SNAIL, SLUG, TWIST, FIBRONECTIN, VIMENTIN,* and *N-CADHERIN*, by qRT–PCR and RT–PCR*.* The results showed that iOVCAR-3-OSKM cells have high expression levels of mesenchymal markers (Fig. 3F, Additional file 6: Figure S5). In addition to its connection with chemoresistance, EMT allows epithelial cells to lose polarity and cell–cell adhesion, which confers migratory and invasive properties. Hence, we used Transwell assays for cell mobility determination. The number of migrated cells was significantly higher in iOVCAR-3-OSKM cells than in parental OVCAR-3 cells (Fig. 3G). In our study, cell mobility was obviously higher in iOVCAR-3-OSKM cells.

### iOVCAR-3-OSKM preserves ovarian lineage differentiation abilities and mimics histological features that indicate a poor prognosis

**Fig. 4:**
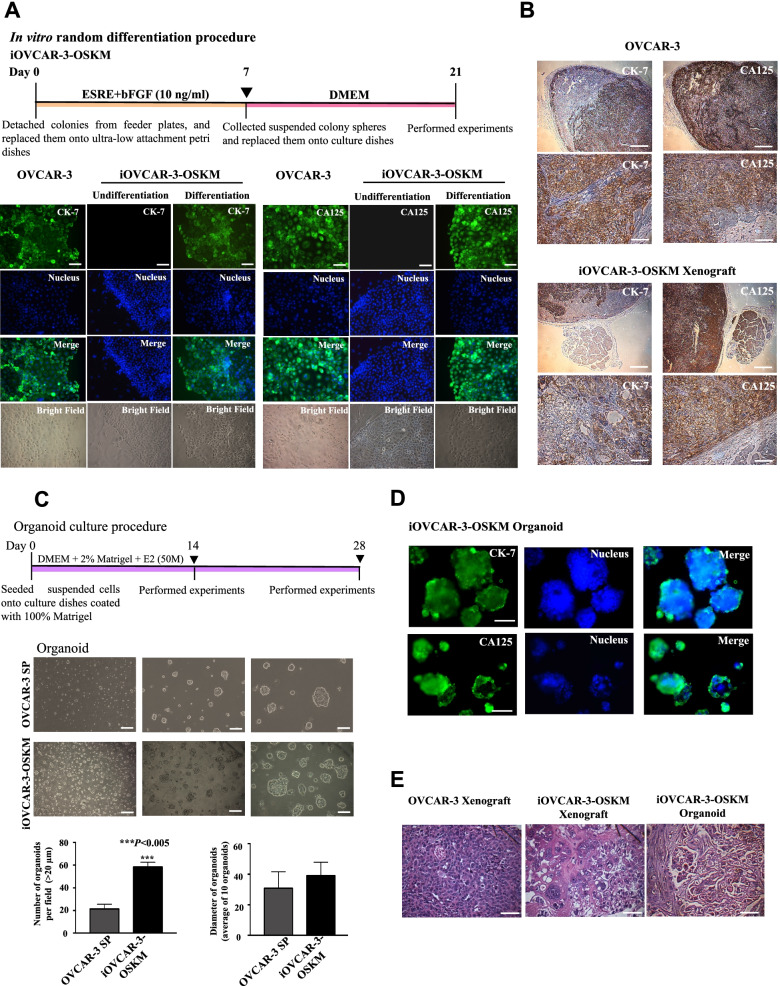
OC lineage-specific differentiation potency. **A** Schematic of the random differentiation procedure and determination of lineage-specific differentiation abilities in vitro*.* IF staining was performed with CK7 and CA125 antibodies under both nondifferentiation and differentiation conditions in iOVCAR-3-OSKM cells. Scale bars: 50 µm. **B** Determination of lineage-specific differentiation in vivo*.* IHC staining was used to assess tumors derived from iOVCAR-3-OSKM cells in NOD-SCID mice. Xenografts of iOVCAR-3-OSKM cells showed both CK7 and CA125 expression. The upper panels show IHC images at 40X magnification (scale bars: 250 µm), and the lower panels show IHC images at 200X magnification (scale bars: 50 µm). **C** Schematic of the organoid culture procedure. A total of 3 × 10^5^ single cells were suspended in medium containing 2% Matrigel with 50 nM E2 and seeded onto culture dishes coated with 100% Matrigel for organoid 3D culture for two weeks or four weeks. The number (> 20 µm organoids were counted under 100X magnification) and diameter (10 organoids were measured under 200X magnification) of two-week organoids were calculated. Scale bars (from left to right): 250 µm; 100 µm; 50 µm. Error bars indicate SD. *P < 0.05, Student’s t test. **D** Two-week organoids were fixed, and IF staining was performed. iOVCAR-3-OSKM cells formed organoids and expressed CK-7 and CA125. Scale bars: 50 µm. **E** H&E staining of xenografts and four-week organoid cultures. Xenograft tumors and organoids were collected and embedded in paraffin. Sections were stained with H&E. Scale bars: 25 µm

Maintenance of stemness is crucial to CICs. Conversely, the capability of cells to differentiate and recapitulate lineage-specific cancer tissues is also a critical characteristic of CICs. To assess the lineage differentiation ability of iOVCAR-3-OSKM cells, we performed an in vitro differentiation procedure and determined the expression levels of CK7 and CA125, which are two widely used OC biomarkers. According to our results, iOVCAR-3-OSKM cells expressed both CK-7 and CA125 after 21 days of differentiation, while undifferentiated cells did not (Fig. 4A). To further determine the lineage differentiation ability in vivo, we histologically assessed xenografts derived from parental OVCAR-3 and iOVCAR-3-OSKM cells. Consistent with the results of the in vitro differentiation assay; the IHC staining indicated that iOVCAR-3-OSKM xenografts presented significant CA125 and CK7 expressions under in vivo differentiation conditions (Fig. 4B).

Then, we tried to establish organoids from iOVCAR-3-OSKM cells to be a more precise modeling for high-throughput screening. Briefly, 3 × 10^5^ single cells were suspended in medium containing 2% Matrigel with 50 nM E2 and seeded onto 100% Matrigel-coated culture dishes. We noticed organoid formation after seven days in culture. Compared to SP cells, iOVCAR-3-OSKM cells were capable of forming more organoids after two weeks of organoid culture (Fig. 4C). IF staining also showed CK-7 and CA125 (OC marker) expression in iOVCAR-3-OSKM organoids (Fig. 4D). We extended the organoid culture to four weeks for histological examination. H&E staining showed that iOVCAR-3-OSKM organoids successfully represented malignant OC histological features, which was impossible in 2D cell cultures (Fig. 4E). Intriguingly, we observed that the histological features of organoids were similar to those of iOVCAR-3-OSKM xenografts. The pathological features of parental OVCAR-3 and iOVCAR-3-OSKM xenografts were dissimilar. Xenografts generated from OVCAR-3 cells predominantly consisted of homogeneous and monotonous expansions of uniform dysplastic cells. However, xenografts derived from iOVCAR-3-OSKM cells showed cell diversity and glandular structures similar to those observed in histological subtypes of clear cell carcinoma (CCC) and endometrioid carcinoma (EC); these are also consistent with our experience upon observing in some of clinical samples of EOC patients with drug resistance or recurrent disease (Fig. 4E) and are suggested to be poor prognostic factors in advanced EOC [36]. Heterogeneity is a hallmark of HGSOC. Compare to OVCAR-3 cell line, the histology of iOVCAR-3-OSKM xenograft displayed more diversity. These findings inferred that in comparison with cell lines, iCICs are likely more suitable for preclinical modeling.

Organoids provide a powerful ex vivo model that rectifies the shortcomings of cell lines and fills the gap between complicated but physiologically relevant xenografts; and our results demonstrated the feasibility of establishing personalized iCIC and iCIC organoids for high-throughput drug screening for OC treatment.

## Discussion

Recently, CICs have attracted attention in cancer research. Patients with OC show high mortality rates due to the development of resistance to chemotherapy and subsequent relapse, events in which CICs are considered to be key “seeds”. Therapeutic strategies targeting CICs are the focus for developing effective anticancer therapies. However, the rarity of CICs in clinical samples and difficulties in their isolation limit research on OC and the discovery of novel therapies. Conventional methods for obtaining CICs are imperative for overcoming OC.

CICs can be separated by combining specific biomarkers that are mostly located on the cell surface by fluorescence-activated cell sorting (FACS) and magnetic-activated cell sorting. OCICs have been identified and isolated according to the expression of different biomarkers, such as CD133 [25], CD117 [26], CD44 [28], or CD24 [27]. Other techniques, such as SP isolation and sphere-formation assays, have also been used [37]. However, the prerequisite of the instrument; the requirements for primary culture, specificity and purity; toxic effects during isolation; and the rarity of CICs in clinical samples limit progress toward the development of CIC targeted therapy. Relative to previous methods, our novel technique evades the instability during the sorting procedure, which not only makes it suitable for setting up a standard operation procedure (SOP) but also provides a feasible way to obtain OCICs more effectively and in greater quantity.

Tumor development and iPSC generation are highly comparable processes with strikingly similar characteristics [12]. Cellular plasticity is inherent in tumor evolution and yields cells that acquire a stem cell‐like phenotype. Evidence suggests that there is a link between pluripotency and cancer; for example, the activation of signaling pathways and genes essential for embryonic-like development during tumor progression and the differentiation ability of tumor cells may be factors [38].

In our data, high OCT4 expression was correlated with worse OS; co-expression with more pluripotent markers was correlated with even worse OS. It is well known that *OSKM* factors are critical for the self-renewal property of stem cells and are enriched in CICs in several cancer types. These factors have also been individually reported to be correlated with malignant behavior and a poor prognosis in various cancers [39–42]. The procedure for generating induced pluripotent stem cells (iPSCs) through ectopic expression of three transcription factors (OCT3/4, SOX2 and KLF4) with or without c-MYC is well established in inducing embryonic stem cells (ESCs) or neural stem cells (NSCs) in vitro. These genes have the abilities to induce various types of stemness in somatic cells under different cell culture conditions. In the previous, several studies have reported the feasibility using at least three Yamanaka factors, OSK, to induce CIC properties in cancer cells. In our experience, we ever choose minimal three factors, OSK, to induce the CIC properties in our OC cells. However, we found that the expressions of c-MYC are distinct in various OC cells. Thus, we finally determined using the four Yamanaka factors, OSKM, to induce our ovarian cancer initiating cells. Moreover, we choose an integration-free SeV gene delivery system to perform the OSKM transduction, this method can help us generating transgene-free and vector-free cells; that is more suitable for the implementation of good manufacturing practice (GMP)-compliant protocols and will be crucial for increasing application safety and fulfilling the legal requirements for clinical application.

To confirm our standard protocol (SOP) for establishing iOCICs by OSKM transduction, we also used another cell line CA5171 to generate iCA5171-OSKM by the transduction SOP that we have set up. CA5171 was a ovarian cancer cell line that derived from a chemotherapy-naive, high-grade undifferentiated ovarian carcinoma [43]. Follow our procedure, iCA5171-OSKM could also be expandable and exhibited stemness characteristics with elevated pluripotent genes, *OCT4, SOX2, C-MYC, KLF4*, and *NANOG*, with strong sphere-forming ability and lineage differentiation ability (Additional file 9: Figure S8).

For lineage differentiation determination, in this study, we used two widely used OC biomarkers, CK7 and CA125 to confirm the OC lineage differentiation. CA125 is a glycoprotein that can be detected by epithelial ovarian cancer antigen, which is widely used in the diagnosis of OC. However, subsequent studies have found that the detection of CA125 has a high false positive rate, and it also has different increasing degrees in other cancers. Therefore, simultaneously detection with other tumor indicators, such as CK7, CK20, ER, PR, C-erbb2, and P-gp was helpful to improve the sensitivity and specificity. Among the mentioned OC tumor markers, CK7 and CA125 have the highest positive rate in malignant OC tissues compared with benign ovarian cancer tissues. Studies also shown that patients with negative expressions of CA125 and CK7 had significantly longer recurrence- free survival (RFS) time than those with positive expressions [44]. Thus, using CA125 and CK7 as malignant OC marker was practical for early diagnosis and lineage determination.

This model is convincing and exhibits characteristics consistent with those of previous studies. Several studies have reported that the proliferation rate of CICs is slower than that of mature cancer cells [45]. CICs predominantly stay in a quiescent state of the cell cycle and re-enter the cell cycle in response to microenvironmental regulation. Conventional therapies have been designed to target proliferating, mature cancer cells, while CICs are mostly quiescent and poorly differentiated. Consequently, they easily evade chemotherapy, producing chemo-resistant recurrent disease [32]. Our results are consistent with those studies on pancreatic cancer, which revealed that a subpopulation of slow proliferating cells in pancreatic adenocarcinoma cell lines exhibited increased tumorigenic and invasive potential in vivo [46]. This implies that external stimuli may trigger cell cycle re-entry of CICs in the quiescent state.

EMT has also been reported in several cancers as a key step to metastatic tumor cell progression and chemoresistance [47]. During EMT, cells acquire mesenchymal properties, which promote invasion into the extracellular matrix, resulting in further dissemination and metastasis. Previous reports indicate the existence of elliptical fibroblast-like cells in HGSOC. These cells display stem cell characteristics and pluripotency and may invade ovarian tissue by transforming from round cells into elongated mesenchymal-like cells with protrusions [48]. Our results are also consistent with those of previous reports; a majority of the enrichment pathways in our gene analysis results are associated with the regulation of ECM and transporter activity.

The exact mechanisms of the initiation of OC remain unclear. Different research groups have discovered CICs in clinical samples, as evidenced by the spontaneous formation of tumor-like structures in vitro. Small tumor-initiating stem cells include NANOG-positive cells in situ in ovaries, and groups of borderline ovarian tumor and EOC cells have been observed [48, 49]. A similar population of larger round cells positively stained for mesenchymal (vimentin) and pluripotency (NANOG, SSEA-4, and SOX2) markers was also observed. Different CICs observed in ovarian tumor tissue support the legitimacy of the CIC theory and indicate the presence of different types of CICs in OC. Hence, the establishment of a CIC model with the ability to mimic the disease precisely is critical to the success of cancer therapy.

Moreover, the unfavorable prognosis of EOC is due to a combination of late, usually advanced-stage, diagnosis and frequent relapse due to chemoresistance. Despite medical progression during the past decade, the mechanisms underlying EOC pathobiology are still poorly understood, and therapeutic efficiency and patient survival rates remain low. Most studies have been performed using cancer cell lines; conventional 2D cell culture has long been established and allows for rapid and reliable growth of cancer cells. However, the technique does not faithfully recapitulate the clinical nature of EOC and the histopathological and molecular phenotype of the tumor of origin, and thus lacks clinical translatability [20]. Although patient tumor-derived xenografts can better mimic the original tumor, limitations, such as ethical controversy and inefficient engraftment, are hard to avoid [50]. Therefore, a more efficient, feasible method for establishing appropriate experimental and preclinical EOC models is required.

iCICs have recently received increasing attention because they offer a new paradigm in cancer modeling. In our knowledge, CICs are rare population in tumor mass and usually hard to be isolated from clinical samples. Since debulking surgery (PDS) following with chemotherapy or neoadjuvant chemotherapy (NACT) combined with delayed interval debulking surgery are the standard guideline for advance EOC treatment. It is easy for doctors or scientists to isolate primary tumor cells from the tissues. Thus, the protocol we established in this study provides a more effective way to obtain greater quantity of OCICs using clinical samples. On the other hand, patients with different genomic backgrounds may display varied clinical phenomena. Our method can customize the iOCICs according to each patient's genetic background. Combine with the organoid 3D-culture technology, we provide a powerful tool for establishing an efficient platform that will improve preclinical research on OC by recapitulating its clinical features under convenient cultivation conditions. This highlights the necessary for generating patient-specific iCICs and cancer organoids; that will revolutionize the approach to personalized medicine including cancer modeling, drug screening and development, autologous/allogenic cell therapy, and precision medicine. Studies on reprogramming cancer cells to harness the versatility of iCICs for cancer research are essential for advancing the knowledge on tumor initiation, interactions in microenvironments, and epigenetic changes that contribute to the development of cancer.

## Conclusions

**Fig. 5:**
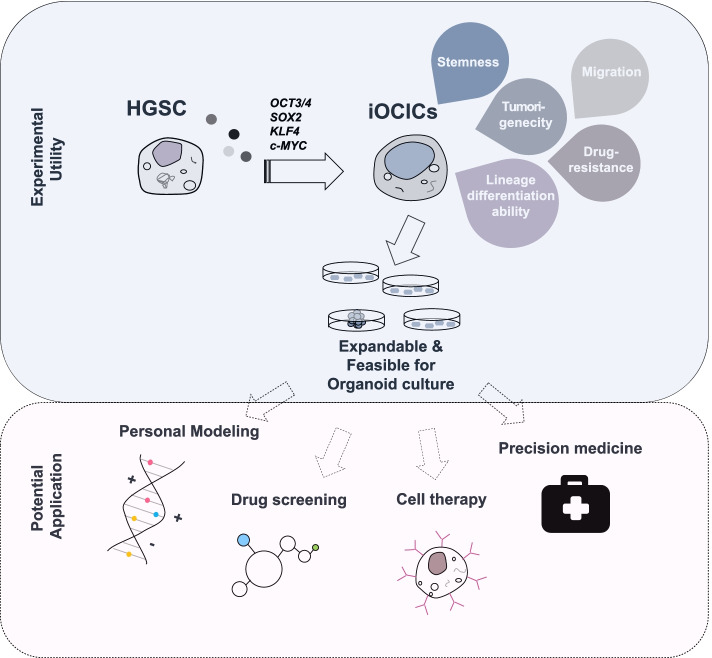
The graphic summary of the iOVCAR-3-OSKM model and its experimental utility and potential applications. iOVCAR-3-OSKM cells form an iCIC model, which is expandable, differentiable, and capable of establishing organoids. This model provides valuable potential as a personalized model for clinical therapy, the establishment of drug screening platforms, the development of cell therapies, and the advancement of precision medicine

We successfully generated an expandable iOCIC model by transducing these cells with four defined factors using an integration-free SeV gene delivery system. This model is expandable, differentiable, and capable of establishing organoids, which is not only helpful in overcoming the limitations of sampling but also provides valuable potential as a personalized model for clinical therapy and is suitable for the implementation of GMP-compliant protocols, increasing the safety of the application and addressing the legal requirements for clinical application (Fig. 5).

Our study could also facilitate the assessment of the underlying mechanisms of OC and enable the establishment of drug screening platforms, the development of better targeting strategies and less toxic drug and cell-based therapies, and the advancement of precision medicine.

## Supplementary Information

**Additional file 1:** **Table S1.** Antibody details. **Table S2.** Oligonucleotide primer sequences. **Table S3.** Summary of induced ovarian cancer initiating cell (iOCIC) generation. **Table S4.** The tumor incidence in the dose test in vivo. **Table S5.** Baseline characteristics of EOC patients in tissue array. **Table S6.** OCT4, SOX2 and NANOG expression in EOC tumor tissues of tissue array. **Table S7.** Co-expression of OCT4, SOX2 and NANOG in EOC tumor tissues of tissue array. **Table S8.** Expression of OCT4, SOX2 and NANOG in eight HGSOC tumor tissues of tissue array.

**Additional file 2:** **Figure S1.** The correlation of the numbers of highly expressed markers with the OS in OC patients.Statistical analysis was carried out using SPSS 22.0 (IBM Corp., Armonk, NY, USA). Kaplan–Meier curves were used to evaluate the correlation of the number of highly expressed markers with OS. Comparisons of two groups were made by the log-rank test. *P ≤ 0.05 was considered to indicate significance.

**Additional file 3:** **Figure S2**. Protein expression of OCT4, TRA1-60, NANOG*, *and CD133 in day 7 iOVCAR-3-OSKM colonies. Colonies were cultured on feeder cells for 7 days. IF was performed with OCT4, TRA1-60, NANOG*, *and CD133 primary antibodies.Scale bars: 50 µm.

**Additional file 4:** **Figure S3.** Protein expression of markers previously reported to be related to OCICs. Target markers were determined by FCM analysis (*n* = 3). Error bars indicate SD. *P < 0.05, Student's *t test*.

**Additional file 5:** **Figure S4.** The proliferation rate of iOVCAR-3-OSKM cells was slower than that of parental cancer cells.Proliferation assay of iOVCAR-3-OSKM cells compared to OVCAR-3 cells. A total of 2 × 10^5^ cells were plated onto a six-well culture plate. The cells were harvested after plating for 24 hours, 48 hours, and 72 hours, andcells were counted. The number of iOVCAR-3-OSKM cells was less than that of OVCAR-3 cells (*n* = 3). Error bars indicate the SD. *P < 0.05, **P < 0.005, Student’s *t test.*

**Additional file 6:** **Figure S5.** The expression of EMT-related genes.qRT–PCR was performed to assess the expression of previously reported EMT-related genes. mRNA expression levels were normalized to those of *GAPDH. *Relative expression levels compared to those of parental cells are shown (*n* = 3). Error bars indicate SD. *P < 0.05, **P < 0.005, Student’s *t test.*

**Additional file 7:** **Figure S6.** Uncropped gel of Figure 2C. Pluripotent gene expression and SeV silencing in iOVCAR-3-OSKM clones. Expression of endogenouspluripotent genes (*OCT4, SOX2, KLF4, and NANOG*), *SeV*, and the housekeeping gene *GAPDH*. cDNA of human ESCs (H9 cell line) and a human induced pluripotent cell line (iPBMCF) were used as positive controls. Neg Ctl: negative control (PCR mixture without cDNA).

**Additional file 8:** **Figure S7.** Uncropped gel of Figure 3F. The expression of EMT-related genes. The endogenous EMT-related genes and the housekeeping gene GAPDH were amplified and separated by electrophoresis. Neg Ctl: negative control (PCR mixture without cDNA).

**Additional file 9:** **Figure S8.** Establishment of the iCA5171-OSKM model, which exhibited stemness and cancer-initiating cell properties. **(A)**Schematic of the transduction procedure and establishment of iOCICs. CA5171 cells were infectedwith SeV containing four factors (*OSKM*) at day 0 and replated onto MEF dishes. Tra-1-60 positive colonies were picked up on day 21 and day 28. Scale bars: 200 µm. **(B)**Pluripotent gene expression and SeV silencing in iCA5171-OSKM clones. Expression of endogenous pluripotent genes (*OCT4, SOX2, KLF4, and NANOG*), *SeV*,and the housekeeping gene *GAPDH*. NC: negative control (PCR mixture without cDNA). The samples derive from the same experiment and gels were processed in parallel. **(C)**Protein expression of OCT4, TRA1-60, NANOG, and CD133 in day 7 iCA5171-OSKM colonies. Colonies were cultured on feeder cells for 7 days. IF was performed with OCT4, TRA1-60, NANOG, and CD133 primary antibodies. Scale bars: 50 µm. **(D)** IF staining was performed with CK7 and CA125 antibodies under differentiation conditions in iCA5171-OSKM cells. Scale bars: 50 µm. **(E)** Sphere-formation ability in vitro. A total of 10^5^cells were plated on low attachment dishes and cultured for 14 days. The numbers of spheroids (≥ 25 µm) were counted under a microscope.  The error bars indicate the SD. Scale bars: 200 µm.

**Additional file 10:** **Figure S9.** Expression of pluripotent genes and the correlation with OS in public OC dataset. **(A) **The analysis of dataset from UCSC XENA browser (http://xena.ucsc.edu). Correlation of each single marker with OS in 376 patients with primary OC from GDC TCGA Ovarian Cancer (OV) cohort. **(B) **Thebox plot of gene expression level with histologic grade. *P < 0.05 was consideredsignificant.

**Additional file 11:** **Figure S10**. Array Comparative Genomic Hybridization (aCGH).aCGH was performed with OVCAR-3 and iOVCAR-3-OSKM for genome-wide screening. aCGH analysis confirmed a 45, XX karyotype of OVCAR-3 and iOVCAR-OSKM, and the consistent detectable geneticalteration.

**Additional file 12: Figure S11.** Uncropped gel of Figure S8B. Pluripotentgene expression and SeV silencing in iCA5171-OSKM clones. Expression of endogenouspluripotent genes (*OCT4, SOX2, KLF4,* and *NANOG*), *SeV*, and the housekeeping gene *GAPDH*. Neg Ctl: negative control (PCR mixture without cDNA).

**Additional file 13: Figure S12.** Expression of pluripotent genes and the correlation with OS in an EOC tissue array. **(A)** Correlation of each single marker with OS in patients with EOC. Statistical analysis was carried out using SPSS 22.0 (IBM Corp., Armonk, NY, USA). Kaplan–Meier curves were used to evaluate the correlation of highly expressed markers with OS. Comparisons of two groups were made by the log-rank test. *P < 0.05 was considered significant. **(B)** The correlation of the numbers of highly expressed markers with the OS in patients with EOC. Kaplan–Meier curves were used to evaluate the correlation of the number of highly expressed markers with OS. Comparisons of two groups were made by the log-rank test. *P < 0.05 was considered to indicate significance.

**Additional file 14: Figure S13.** Expression of pluripotent genes and the correlation with OS in HGSOC cases of an EOC tissue array. **(A)** Correlation of each single marker with OS in eight patients with HGSOC. Statistical analysis was carried out using SPSS 22.0 (IBM Corp., Armonk, NY, USA). Kaplan–Meier curves were used to evaluate the correlation of highly expressed markers with OS. Comparisons of two groups were made by the log-rank test. *P < 0.05 was considered significant. **(B)** The correlation of the numbers of highly expressed markers with the OS in eight patients with HGSOC. Kaplan–Meier curves were used to evaluate the correlation of the number of highly expressed markers with OS. Comparisons of two groups were made by the log-rank test. *P < 0.05 was considered to indicate significance.

## Data Availability

All data generated or analyzed during this study are included in this published article [and its supplementary information files]. The RNA-seq datasets generated and analyzed during the current study are available in the GEO repository, GSE201206. Should there be any further requests and questions, the data used and/or analyzed during the current study are available from the corresponding author on reasonable request. All gels used in the figures followed the digital image and integrity policies.

## Abbreviations

ABC: ATP-binding cassette
BP: Biological process
CC: Cellular component
CCC: Clear cell carcinoma
CIC: Cancer-initiating cells
CSC: Cancer stem cells
CSLC: Cancer stem-like cells
DEG: Differentially expressed genes
DMEM: Dulbecco's modified Eagle's medium
EC: Endometrioid carcinoma
ECM: Extracellular matrix
EMT: Epithelial-mesenchymal transition
EOC: Epithelial ovarian cancer
ESCs: Embryonic stem cells
FACS: Fluorescence-activated cell sorter
FBS: Fetal bovine serum
FCM: Flow cytometry
GFR: Growth factor reduced
GO: Gene Ontology
HGSOC: High-grade serous ovarian carcinoma
HOSE: Human ovarian surface epithelial
IBD: Inflammatory bowel disease
IF: Immunofluorescent
IHC: Immunohistochemistry
iPSCs: Induced pluripotent stem cells
iCICs: Induced cancer-initiating cells
KEGG: Kyoto Encyclopedia of Genes and Genomes
KLF4: Kruppel-like factor 4
KOSR: Knockout serum replacement
MEF: Mouse embryonic fibroblast
MF: Molecular function
MP: Main population
OC: Ovarian cancer
OCIC: Ovarian cancer-initiating cell
OCT3/4: Octamer-binding transcription factor 3/4
OS: Overall survival
OSKM: *OCT3/4, SOX2, KLF4,* And *c-MYC*
PBMCs: Peripheral blood mononuclear cells
PCR: Polymerase chain reaction
PI: Propidium iodide
SOX2: SRY-box transcription factor 2
SP: Side population
VM: Verapamil
