# Supplementary material for: An experimental model for ovarian cancer: propagation of ovarian cancer initiating cells and generation of ovarian cancer organoids
Source: BMC Cancer. 2022 Sep 10;22:967. doi: 10.1186/s12885-022-10042-3 (PMC9463800; doi:10.1186/s12885-022-10042-3)
Supplement: Supplementary file 1 — Additional file 1: Table S1. Antibody details. Table S2. Oligonucleotide primer sequences. Table S3. Summary of induced ovarian cancer initiating cell (iOCIC) generation. Table S4. The tumor incidence in the dose test in vivo. Table S5. Baseline characteristics of EOC patients in tissue array. Table S6. OCT4, SOX2 and NANOG expression in EOC tumor tissues of tissue array. Table S7. Co-expression of OCT4, SOX2 and NANOG in EOC tumor tissues of tissue array. Table S8. Expression of OCT4, SOX2 and NANOG in eight HGSOC tumor tissues of tissue array. [file 12885_2022_10042_MOESM1_ESM.docx]

|  | **Antibody** | **Dilution** | **Company Cat#** |
| --- | --- | --- | --- |
| Pluripotency Marker (IHC) | Mouse anti-OCT4 IgG | 1:20 | Santa cruz Cat# SC-5279 |
| Pluripotency Marker (IHC) | Rabbit anti-SOX2 IgG | 1:250 | Millipore Cat# AB5603 |
| Pluripotency Marker (IHC) | Rabbit Anti-Nanog IgG | RTU | BioSB Cat#BSB3581 |
| Pluripotency Marker (IF/FCM) | Rabbit anti-OCT4 IgG | 1:100 | Abcam Cat#ab200834 |
| Pluripotency Marker (IF/FCM) | Mouse anti-TRA-1-60 IgM | 1:100 | Millipore Cat#MAB4360 |
| Pluripotency Marker (IF/FCM) | Rabbit Anti-Nanog IgG | 1:100 | Abcam Cat#ab21624 |
| OCIC related gene (IF/FCM) | Rabbit anti-CD133 IgG | 1:200 | Abcam Cat#ab19898 |
| OCIC related gene (FCM) | Goat anti-CD117 IgG | 2.5 µg/10^6^ cells | R&D Cat#AF1356 |
| OCIC related gene (FCM) | Mouse anti-CD24 IgG | 2.5 µg/10^6^ cells | Thermo Fisher Scientific  Cat#MA5-11828 |
| OCIC related gene (FCM) | Mouse anti-CD44 IgG | 0.5 𝜇g /10^6^ cells | Abcam Cat#ab6124 |
| OCIC related gene (FCM) | Rat anti-ABCG2 IgG | 1 𝜇g /10^6^ cells | Abcam Cat#ab24115 |
| OCIC related gene (FCM) | Rabbit anti-ALDH1 IgG | 1:500 | Abcam Cat#ab24115 |
| Differentiation Marker (IF/FCM) | Rabbit anti-CK7 IgG | 1:100 | Abcam Cat#ab68459 |
| Differentiation Marker (IF/FCM) | Mouse anti-CA125 IgG | 1:100 | Abcam Cat#ab134093 |
| Secondary antibody | Alexa Fluor 488 donkey anti-rat IgG | 1:250 | Thermo Fisher Scientific  Cat# A-24876 |
| Secondary antibody | Alexa Fluor 488 donkey anti-goat IgG | 1:250 | Thermo Fisher Scientific  Cat# A-11055 |
| Secondary antibody | Alexa Fluor 488 donkey anti-rabbit IgG | 1:250 | Thermo Fisher Scientific  Cat# A-25535 |
| Secondary antibody | Alexa Fluor 488 goat anti-mouse IgG | 1:250 | Thermo Fisher Scientific  Cat# A-25536 |
| Secondary antibody | Alexa Fluor 488 Goat anti-Mouse IgM | 1:250 | Thermo Fisher Scientific  Cat# A-21042 |

**Additional file 1:**

**Table S1. Antibodies details**

**Table S2. Oligonucleotide primer sequences details**

|  | **Target** | **Forward/Reverse primer (5′-3′)** |
| --- | --- | --- |
| Sendai virus detection | *SeV* | F: GGATCACTAGGTGATATCGAGC  R: ACCAGACAAGAGTTTAAGAGATATGTATC |
| Pluripotent marker | *OCT4* | F: TGTACTCCTCGGTCCCTTTC R: TCCAGGTTTTCTTTCCTAGC |
| Pluripotent marker | *SOX2* | F: GCTAGTCTCCAAGCGACGAA R: GCAAGAAGCCTCTCCTTGAA |
| Pluripotent marker | *KLF4* | F: TTCCTGCATGCCAGAGGAGCCC  R: AATGTATCGAAGGTGCTCAA |
| Pluripotent marker | *c-MYC* | F: TAACTGACTAGCAGGCTTGTCG  R: TCCACATACAGTCCTGGATGATGATG |
| Pluripotent marker | *NANOG* | F: CAGTCTGGACACTGGCTGAA R: CTCGCTGATTAGGCTCCAAC |
| EMT genes | *TWIST* | F: TGCATGCATTCTCAAGAGGT  R: GTTTTGCAGGCCAGTTTGAT |
| EMT genes | *SNAIL* | F: GCTCCACAAGCACCAAGAGT  R: ATTCCATGGCAGTGAGAAGG |
| EMT genes | *SLUG* | F: CTTTTTCTTGCCCTCACTGC  R: ACAGCAGCCAGATTCCTCAT |
| EMT genes | *E-CAD* | F: CAATGCCGCCATCGCTTAC  R: ATGACTCCTGTGTTCCTGTTAATG |
| EMT genes | *N-CAD* | F: GACAATGCCCCTCAAGTGTT  R: CCATTAAGCCGAGTGATGGT |
| EMT genes | *VIMENTIN* | F: GAGAACTTTGCCGTTGAAGC  R: TCCAGCAGCTTCCTGTAGGT |
| EMT genes | *FIBRONECTIN* | F: ACCAACCTACGGATGACTCG  R: GCTCATCATCTGGCCATTTT |
| OCIC related genes | *CD117* | F: TGCTTCACAGAAGACCATGC  R: GTGACCAACATGGAGTCGTG |
| OCIC related genes | *CD133* | F: GGACCCATTGGCATTCTC  R: CAGGACACAGCATAGAATAATC |
| OCIC related genes | *CD24* | F: TACCCACGCAGATTTATT  R: AGAGTGAGACCACGAAGA |
| OCIC related genes | *CD44* | F: GACAAGTTTTGGTGGCACG  R: CACGTGGAATACACCTGCAA |
| OCIC related genes | *ABCG2* | F: TATAGCTCAGATCATTGTCACAGTC  R: GTTGGTCGTCAGGAAGAAGAG |
| OCIC related genes | *ALDH1* | F: TGTTAGCTGATGCCGACTTG  R: TTCTTAGCCCGCTCAACACT |
| House-Keeping Gene | *GAPDH* | F: AGCCACATCGCTCAGACACC  R: GTACTCAGCGGCCAGCATCG |

**Table S3. Summary of induced ovarian cancer initiating cell (iOCIC) generation**

| **Parental cell: OVCAR-3, high grade serous epithelial ovarian cancer cell (HGSOC)** | | |
| --- | --- | --- |
| Number of parental cells seeded | Number of early reprograming colonies | Number of established clones |
| 1×10^5^ | >300 | 8 |
| 2×10^5^ | <100 | 1 |

**Table S4.** The tumor incidence in the dose test *in vivo*

|  | **Injected cell number (cells)** | | | | |
| --- | --- | --- | --- | --- | --- |
| **Cell name** | ***10^7^*** | ***10^6^*** | ***10^5^*** | ***10^4^*** | ***10^3^*** |
| **OVCAR-3** | 100% (2/2) | 100% (2/2) | 100% (2/2) | **0% (0/2)** | **0% (0/2)** |
| **iOVCAR-3-OSKM** | 100% (2/2) | 100% (2/2) | 100% (2/2) | **100% (2/2)** | **100% (2/2)** |

| Parameters | | OC patients  n (%), total patients No. 18 |
| --- | --- | --- |
| Age (yrs) | | 54.77±8.42 |
| Histotype | |  |
|  | High-grade serous carcinoma | 8(44) |
|  | Endometrioid adenocarcinoma | 2 (11) |
|  | Mucinous adenocarcinoma | 1(6) |
|  | Adenocarcinoma | 7(39) |
| Differentiation type | |  |
|  | Well differentiated | 0 (0) |
|  | Moderately differentiated | 4 (22) |
|  | Poorly differentiated | 14 (78) |
| T stage | |  |
|  | T1 | 2 (11) |
|  | T2 | 2 (11) |
|  | T3 | 14 (78) |
| N stage | |  |
|  | N0 | 12 (67) |
|  | N1 | 6 (33) |
| M stage | |  |
|  | M0 | 18 (100) |
| TNM stage | |  |
|  | I | 2 (11) |
|  | II | 2 (11) |
|  | III | 14 (78) |

**Table. S5 Baseline characteristics of EOC patients in tissue array**

OC, Ovarian cancer.

EOC, Epithelial ovarian carcinoma

Dara is presented as mean±SD or counts (with or without percentage).

Table. S6 **OCT4, SOX2 and NANOG expression in EOC tumor tissues of tissue array**

| Parameters  (n=18) | OCT4^high^ | OCT4^low^ | SOX2^high^ | SOX2^low^ | NANOG^high^ | NANOG^low^ |
| --- | --- | --- | --- | --- | --- | --- |
| Tumor tissue (%) | 12 (67) | 6 (33) | 13 (72) | 5 (28) | 11 (61) | 7 (39) |

Data are presented as counts (percentage). The histological score (HSCORE) ranged from 0 to 3 and was calculated to assess immunohistochemical staining by the following formula: HSCORE=ΣPi(i) where “pi” represents the percentage of positive cell counts in total cell counts, and “i” represents the intensity). An HSCORE of 0.05 was considered as a threshold to distinguish high expression and low expression.

Table. S7 **Co-expression of OCT4, SOX2 and NANOG in EOC tumor tissues of tissue array**

| Parameters | | OC patients (n=18) |
| --- | --- | --- |
| Patients with no highly expressed marker (%) | | 3 (17) |
| Patients with only one highly expressed marker (%) | | 2 (11) |
| Patients with two highly expressed markers (%) | | 5 (28) |
|  | OCT4^high^ and SOX2^high^ | 3 (17) |
|  | OCT4^high^ and NANOG^high^ | 1 (6) |
|  | SOX2^high^ and NANOG^high^ | 1 (6) |
| Patients with three highly expressed markers (%) | | 8 (44) |

OC, Ovarian cancer.

EOC, Epithelial ovarian carcinoma

Data are presented as counts (percentage)

Table. S8 **Expression of OCT4, SOX2 and NANOG in eight HGSOC tumor tissues of tissue array**

| Parameters | | HGSOC patients (n=8) |
| --- | --- | --- |
| Patients with no highly expressed marker (%) | | 2 (25) |
| Patients with only one highly expressed marker (%) | | 2 (25) |
|  | OCT4^high^ | 0 (0) |
|  | SOX2^high^ | 1 (13) |
|  | NANOG^high^ | 1 (13) |
| Patients with two highly expressed markers (%) | | 0 (0) |
| Patients with three highly expressed markers (%) | | 4 (50) |

OC, Ovarian cancer.

EOC, Epithelial ovarian carcinoma

Data are presented as counts (percentage)
