# Supplementary material for: An experimental model for ovarian cancer: propagation of ovarian cancer initiating cells and generation of ovarian cancer organoids
Source: BMC Cancer. 2022 Sep 10;22:967. doi: 10.1186/s12885-022-10042-3 (PMC9463800; doi:10.1186/s12885-022-10042-3)
Supplement: Supplementary file 2 — Additional file 2: Figure S1. The correlation of the numbers of highly expressed markers with the OS in OC patients.Statistical analysis was carried out using SPSS 22.0 (IBM Corp., Armonk, NY, USA). Kaplan–Meier curves were used to evaluate the correlation of the number of highly expressed markers with OS. Comparisons of two groups were made by the log-rank test. *P ≤ 0.05 was considered to indicate significance. [file 12885_2022_10042_MOESM2_ESM.pdf]

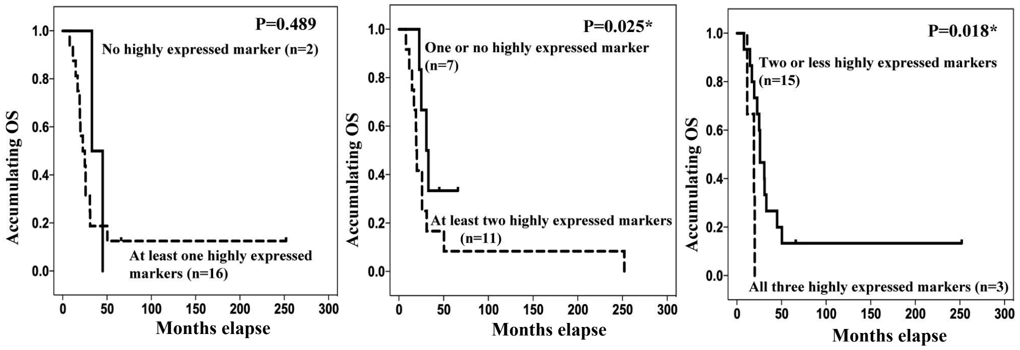

Figure S1. The correlation of the numbers of highly expressed markers with the OS in OC patients. Statistical analysis was carried out using SPSS 22.0 (IBM Corp., Armonk, NY, USA). Kaplan–Meier curves were used to evaluate the correlation of the number of highly expressed markers with OS. Comparisons of two groups were made by the log-rank test. $^*P \leq 0.05$ was considered to indicate significance.
