# Supplementary material for: An experimental model for ovarian cancer: propagation of ovarian cancer initiating cells and generation of ovarian cancer organoids
Source: BMC Cancer. 2022 Sep 10;22:967. doi: 10.1186/s12885-022-10042-3 (PMC9463800; doi:10.1186/s12885-022-10042-3)
Supplement: Supplementary file 3 — Additional file 3: Figure S2. Protein expression of OCT4, TRA1-60, NANOG, and CD133 in day 7 iOVCAR-3-OSKM colonies. Colonies were cultured on feeder cells for 7 days. IF was performed with OCT4, TRA1-60, NANOG, and CD133 primary antibodies.Scale bars: 50 µm. [file 12885_2022_10042_MOESM3_ESM.pdf]

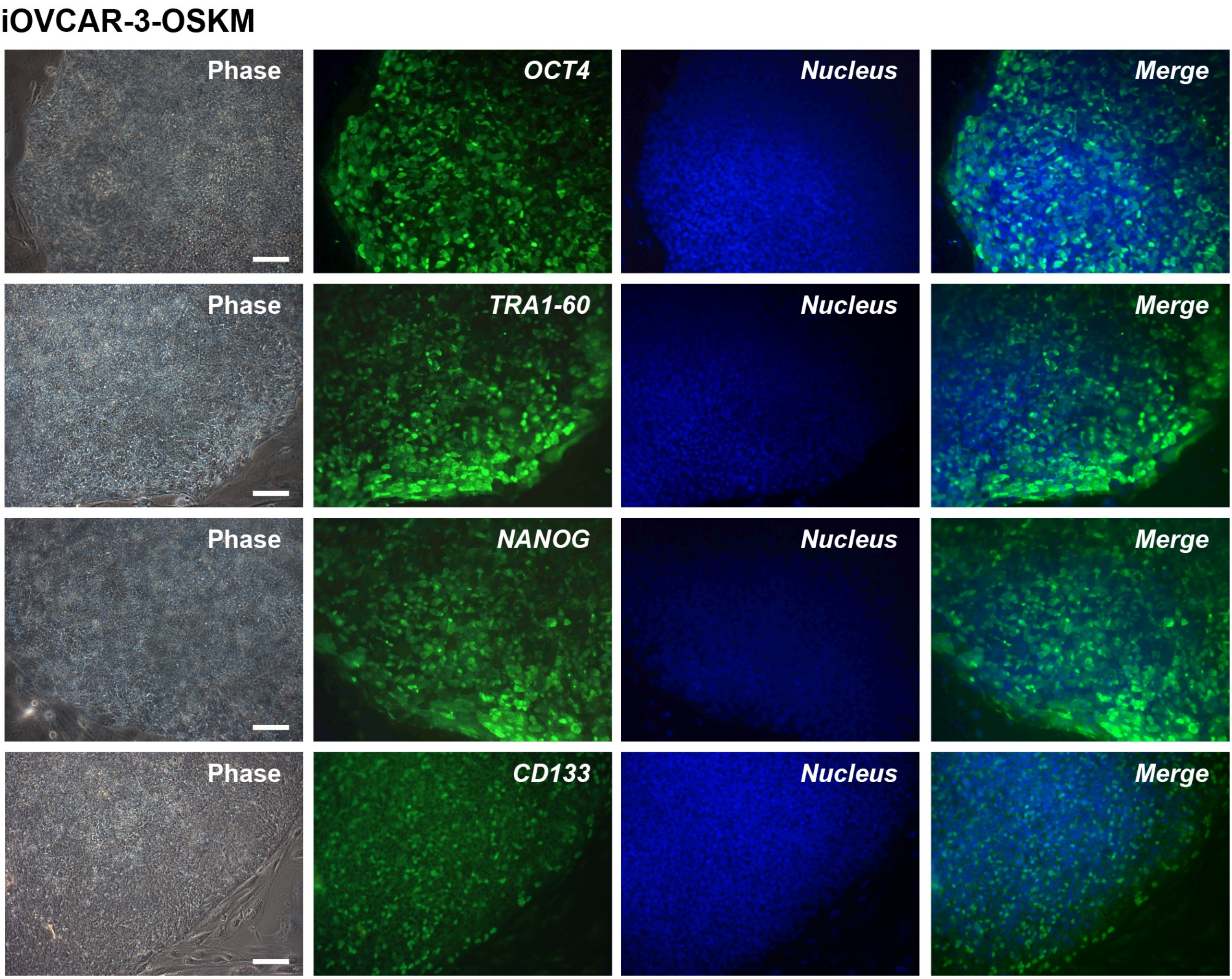

Figure S2. Protein expression of OCT4, TRA1-60, NANOG, and CD133 in day 7 iOVCAR-3-OSKM colonies. Colonies were cultured on feeder cells for 7 days. IF was performed with OCT4, TRA1-60, NANOG, and CD133 primary antibodies. Scale bars: 50 μm.
