# Supplementary material for: An experimental model for ovarian cancer: propagation of ovarian cancer initiating cells and generation of ovarian cancer organoids
Source: BMC Cancer. 2022 Sep 10;22:967. doi: 10.1186/s12885-022-10042-3 (PMC9463800; doi:10.1186/s12885-022-10042-3)
Supplement: Supplementary file 4 — Additional file 4: Figure S3. Protein expression of markers previously reported to be related to OCICs. Target markers were determined by FCM analysis (n = 3). Error bars indicate SD. *P < 0.05, Student's t test. [file 12885_2022_10042_MOESM4_ESM.pdf]

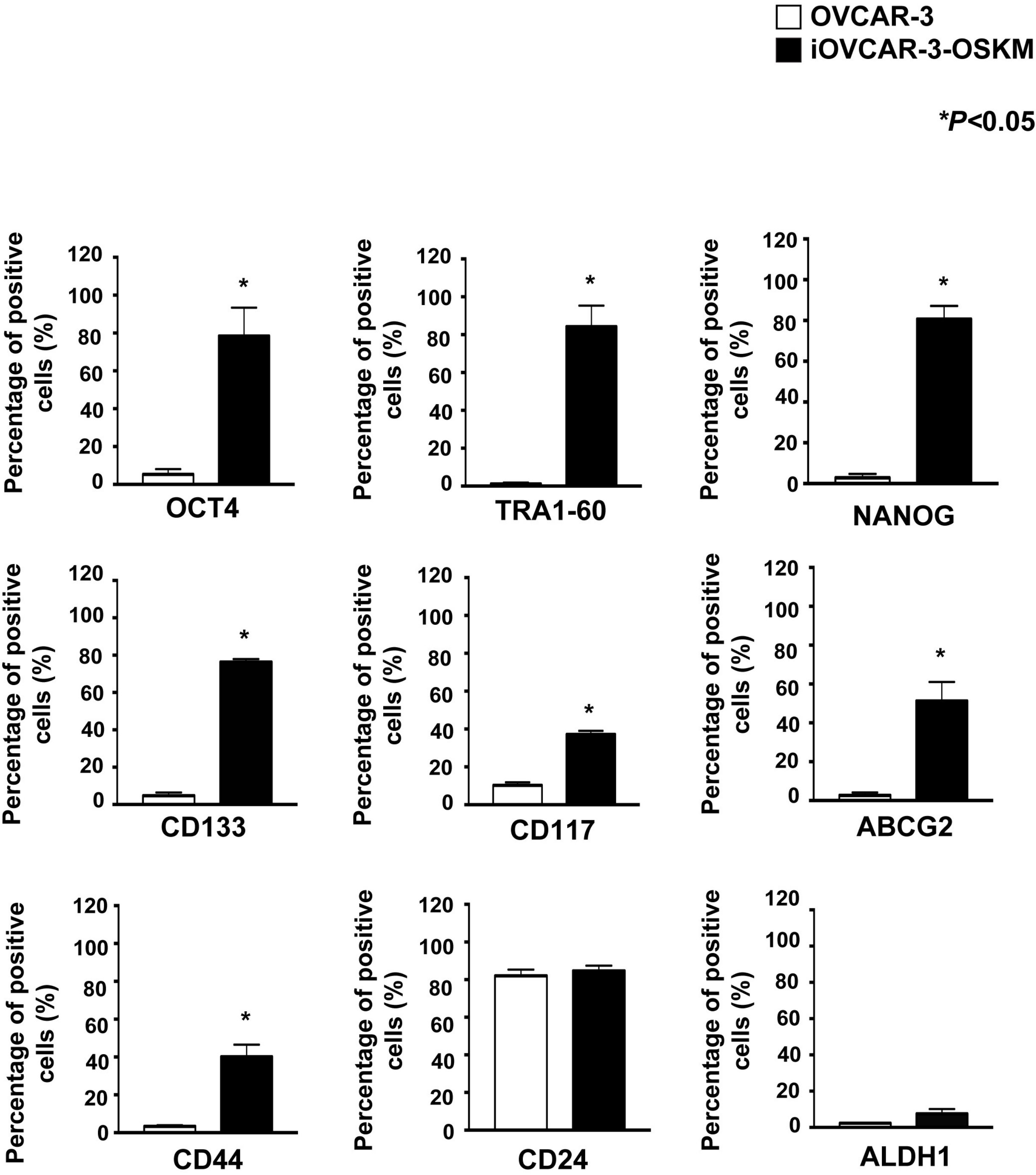

Figure S3. Protein expression of markers previously reported to be related to OCICs. Target markers were determined by FCM analysis (n = 3). Error bars indicate SD. *$P < 0.05$, Student's *t test*.
