# Supplementary material for: An experimental model for ovarian cancer: propagation of ovarian cancer initiating cells and generation of ovarian cancer organoids
Source: BMC Cancer. 2022 Sep 10;22:967. doi: 10.1186/s12885-022-10042-3 (PMC9463800; doi:10.1186/s12885-022-10042-3)
Supplement: Supplementary file 5 — Additional file 5: Figure S4. The proliferation rate of iOVCAR-3-OSKM cells was slower than that of parental cancer cells.Proliferation assay of iOVCAR-3-OSKM cells compared to OVCAR-3 cells. A total of 2 × 10^5^ cells were plated onto a six-well culture plate. The cells were harvested after plating for 24 hours, 48 hours, and 72 hours, andcells were counted. The number of iOVCAR-3-OSKM cells was less than that of OVCAR-3 cells (n = 3). Error bars indicate the SD. *P < 0.05, **P < 0.005, Student’s t test. [file 12885_2022_10042_MOESM5_ESM.pdf]

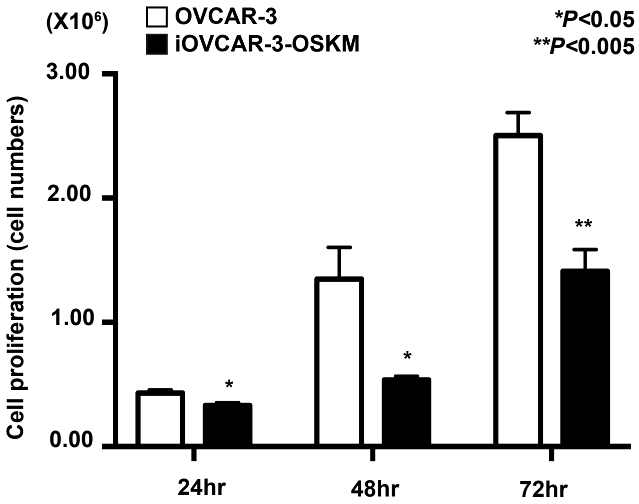

Figure S4. The proliferation rate of iOVCAR-3-OSKM cells was slower than that of parental cancer cells. Proliferation assay of iOVCAR-3-OSKM cells compared to OVCAR-3 cells. A total of 2×10^5 cells were plated onto a six-well culture plate. The cells were harvested after plating for 24 hours, 48 hours, and 72 hours, and cells were counted. The number of iOVCAR-3-OSKM cells was less than that of OVCAR-3 cells (n = 3). Error bars indicate the SD. $*P < 0.05$, $**P < 0.005$, Student's *t test.*
