# Supplementary material for: An experimental model for ovarian cancer: propagation of ovarian cancer initiating cells and generation of ovarian cancer organoids
Source: BMC Cancer. 2022 Sep 10;22:967. doi: 10.1186/s12885-022-10042-3 (PMC9463800; doi:10.1186/s12885-022-10042-3)
Supplement: Supplementary file 6 — Additional file 6: Figure S5. The expression of EMT-related genes.qRT–PCR was performed to assess the expression of previously reported EMT-related genes. mRNA expression levels were normalized to those of GAPDH. Relative expression levels compared to those of parental cells are shown (n = 3). Error bars indicate SD. *P < 0.05, **P < 0.005, Student’s t test. [file 12885_2022_10042_MOESM6_ESM.pdf]

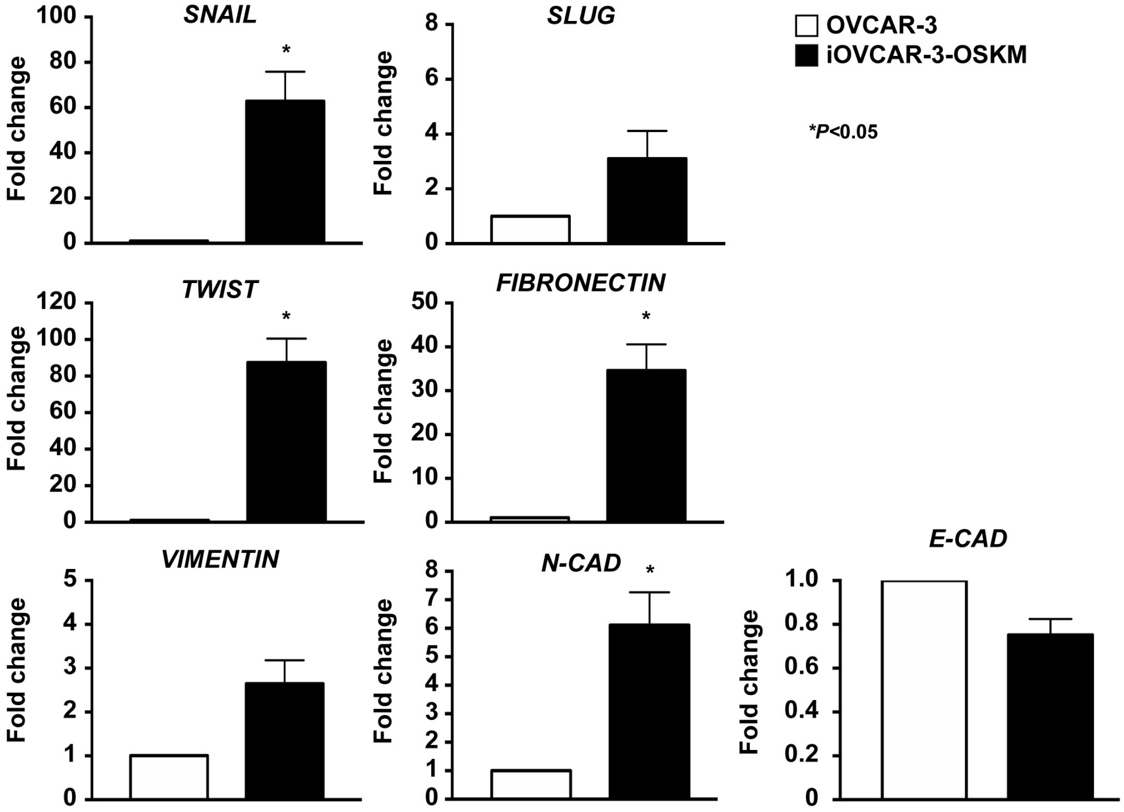

Figure S5. The expression of EMT-related genes. qRT–PCR was performed to assess the expression of previously reported EMT-related genes. mRNA expression levels were normalized to those of GAPDH. Relative expression levels compared to those of parental cells are shown (n = 3). Error bars indicate SD. *$P < 0.05$, **$P < 0.005$, Student's *t test*.
