# Supplementary material for: An experimental model for ovarian cancer: propagation of ovarian cancer initiating cells and generation of ovarian cancer organoids
Source: BMC Cancer. 2022 Sep 10;22:967. doi: 10.1186/s12885-022-10042-3 (PMC9463800; doi:10.1186/s12885-022-10042-3)
Supplement: Supplementary file 7 — Additional file 7: Figure S6. Uncropped gel of Figure 2C. Pluripotent gene expression and SeV silencing in iOVCAR-3-OSKM clones. Expression of endogenouspluripotent genes (OCT4, SOX2, KLF4, and NANOG), SeV, and the housekeeping gene GAPDH. cDNA of human ESCs (H9 cell line) and a human induced pluripotent cell line (iPBMCF) were used as positive controls. Neg Ctl: negative control (PCR mixture without cDNA). [file 12885_2022_10042_MOESM7_ESM.pdf]

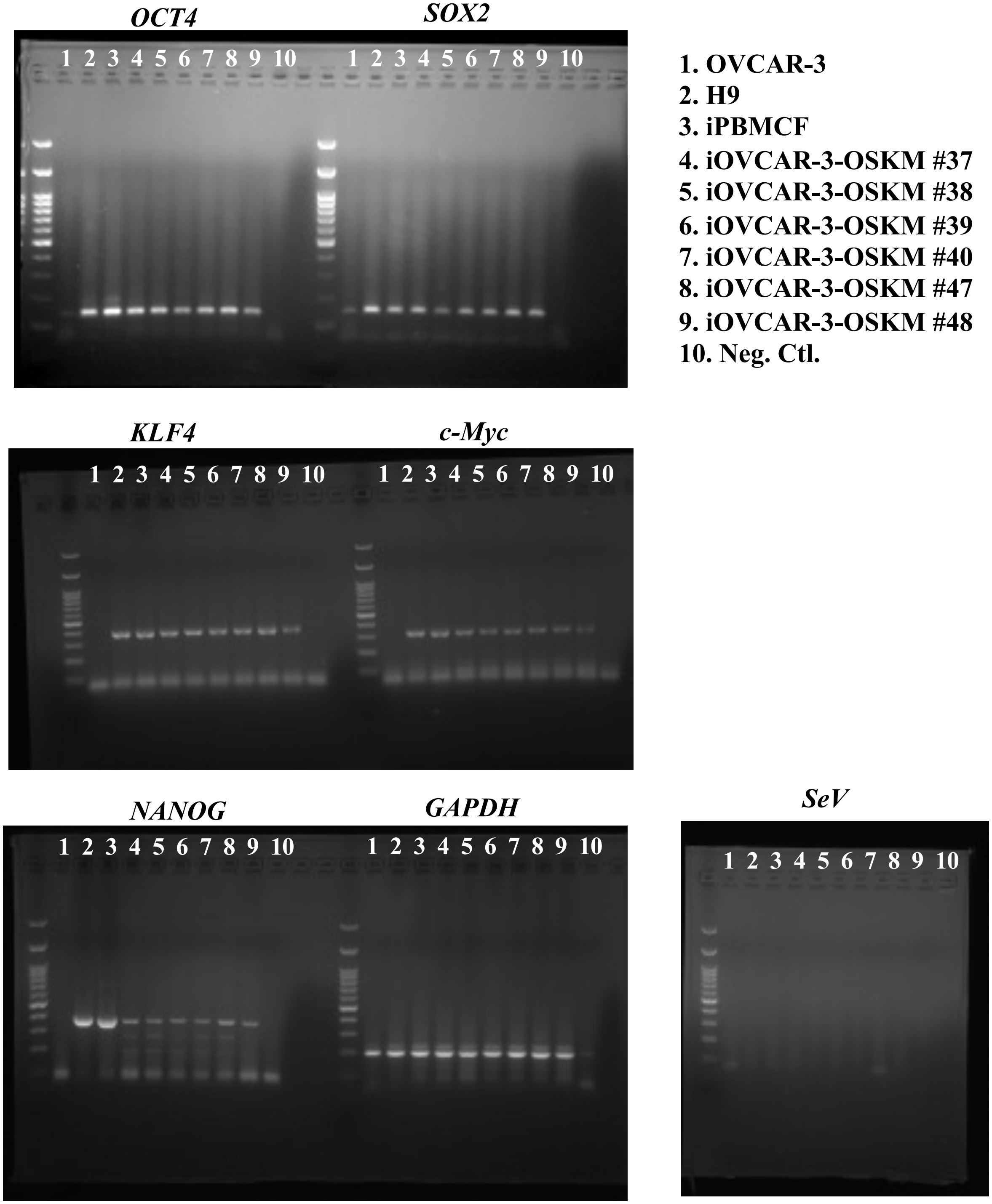

Figure S6. Uncropped gel of Figure 2C. Pluripotent gene expression and SeV silencing in iOVCAR-3-OSKM clones. Expression of endogenous pluripotent genes (*OCT4, SOX2, KLF4, and NANOG*), *SeV*, and the housekeeping gene *GAPDH*. cDNA of human ESCs (H9 cell line) and a human induced pluripotent cell line (iPBMCF) were used as positive controls. Neg Ctl: negative control (PCR mixture without cDNA).
