# Supplementary material for: An experimental model for ovarian cancer: propagation of ovarian cancer initiating cells and generation of ovarian cancer organoids
Source: BMC Cancer. 2022 Sep 10;22:967. doi: 10.1186/s12885-022-10042-3 (PMC9463800; doi:10.1186/s12885-022-10042-3)
Supplement: Supplementary file 8 — Additional file 8: Figure S7. Uncropped gel of Figure 3F. The expression of EMT-related genes. The endogenous EMT-related genes and the housekeeping gene GAPDH were amplified and separated by electrophoresis. Neg Ctl: negative control (PCR mixture without cDNA). [file 12885_2022_10042_MOESM8_ESM.pdf]

1. OVCAR-3
2. iOVCAR-3-OSKM
3. Neg. Ctl.

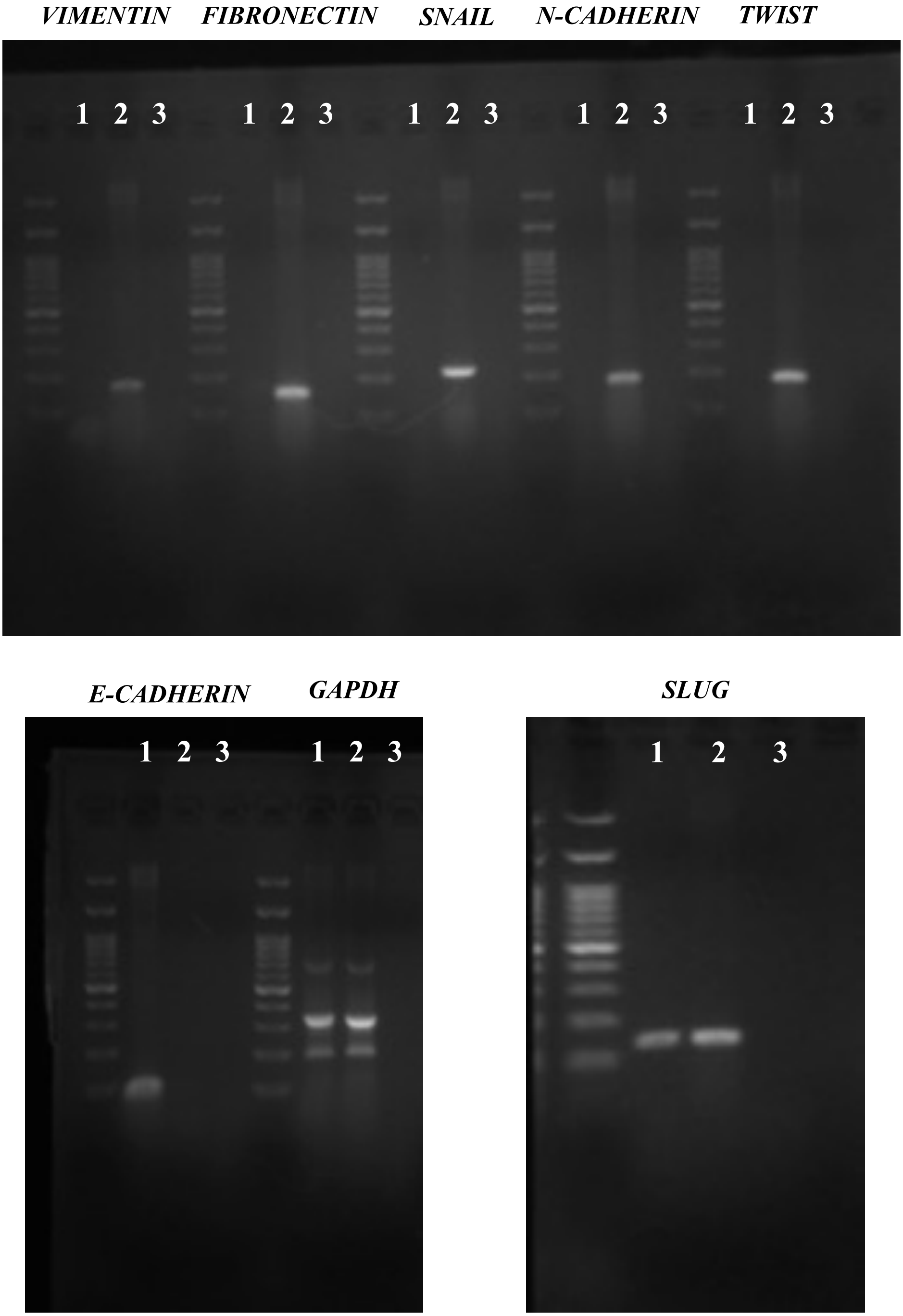

Figure S7. Uncropped gel of Figure 3F. The expression of EMT-related genes. The endogenous EMT-related genes and the housekeeping gene GAPDH were amplified and separated by electrophoresis. Neg Ctl: negative control (PCR mixture without cDNA).
