# Supplementary material for: An experimental model for ovarian cancer: propagation of ovarian cancer initiating cells and generation of ovarian cancer organoids
Source: BMC Cancer. 2022 Sep 10;22:967. doi: 10.1186/s12885-022-10042-3 (PMC9463800; doi:10.1186/s12885-022-10042-3)
Supplement: Supplementary file 10 — Additional file 10: Figure S9. Expression of pluripotent genes and the correlation with OS in public OC dataset. (A) The analysis of dataset from UCSC XENA browser (http://xena.ucsc.edu). Correlation of each single marker with OS in 376 patients with primary OC from GDC TCGA Ovarian Cancer (OV) cohort. (B) Thebox plot of gene expression level with histologic grade. *P < 0.05 was consideredsignificant. [file 12885_2022_10042_MOESM10_ESM.pdf]

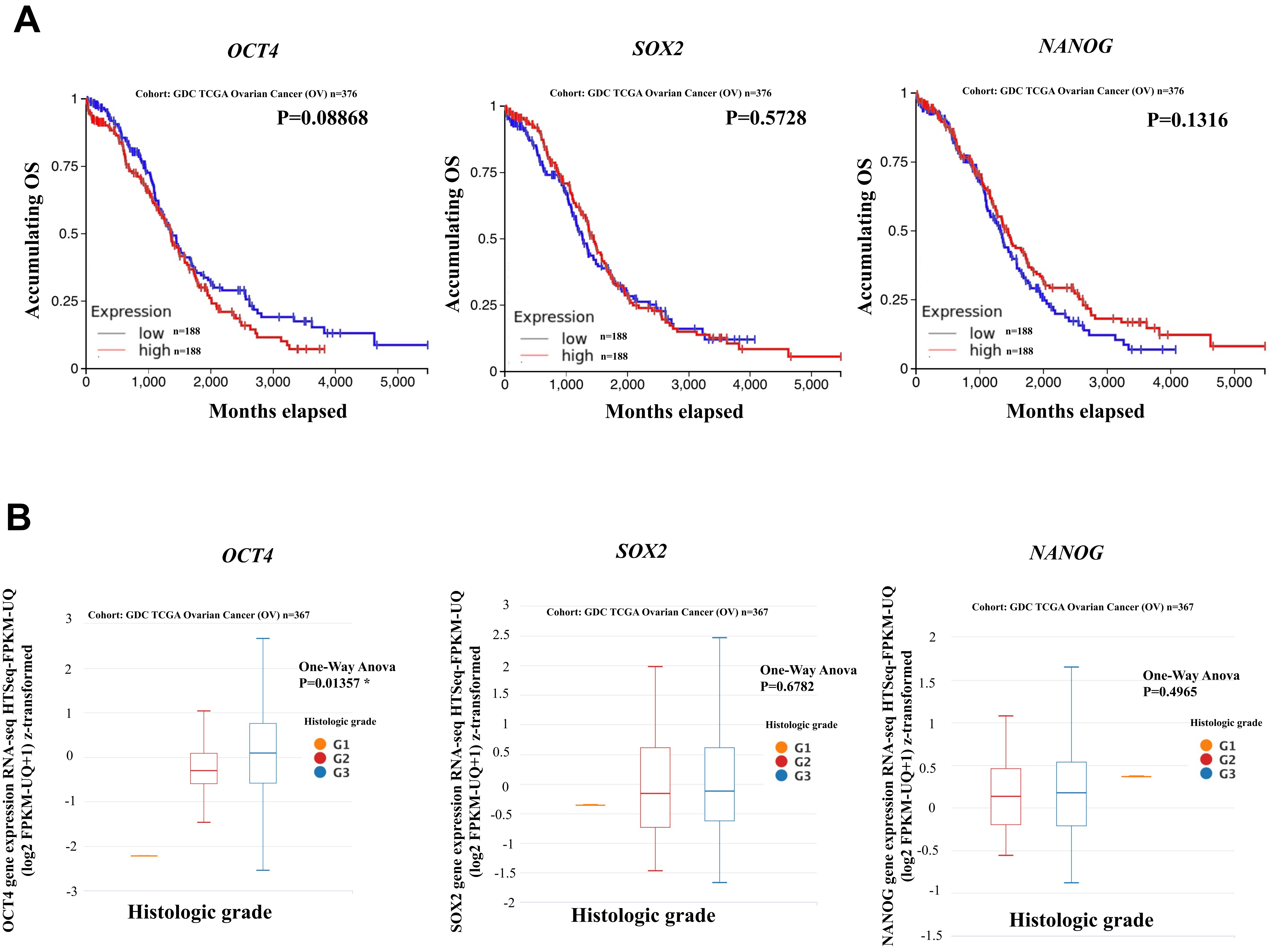

Figure S9. Expression of pluripotent genes and the correlation with OS in public OC dataset. (A) The analysis of dataset from UCSC XENA browser (http://xena.ucsc.edu). Correlation of each single marker with OS in 376 patients with primary OC from GDC TCGA Ovarian Cancer (OV) cohort. **(B)** The box plot of gene expression level with histologic grade. *P ≤ 0.05 was considered significant.
