# Supplementary material for: An experimental model for ovarian cancer: propagation of ovarian cancer initiating cells and generation of ovarian cancer organoids
Source: BMC Cancer. 2022 Sep 10;22:967. doi: 10.1186/s12885-022-10042-3 (PMC9463800; doi:10.1186/s12885-022-10042-3)
Supplement: Supplementary file 11 — Additional file 11: Figure S10. Array Comparative Genomic Hybridization (aCGH).aCGH was performed with OVCAR-3 and iOVCAR-3-OSKM for genome-wide screening. aCGH analysis confirmed a 45, XX karyotype of OVCAR-3 and iOVCAR-OSKM, and the consistent detectable geneticalteration. [file 12885_2022_10042_MOESM11_ESM.pdf]

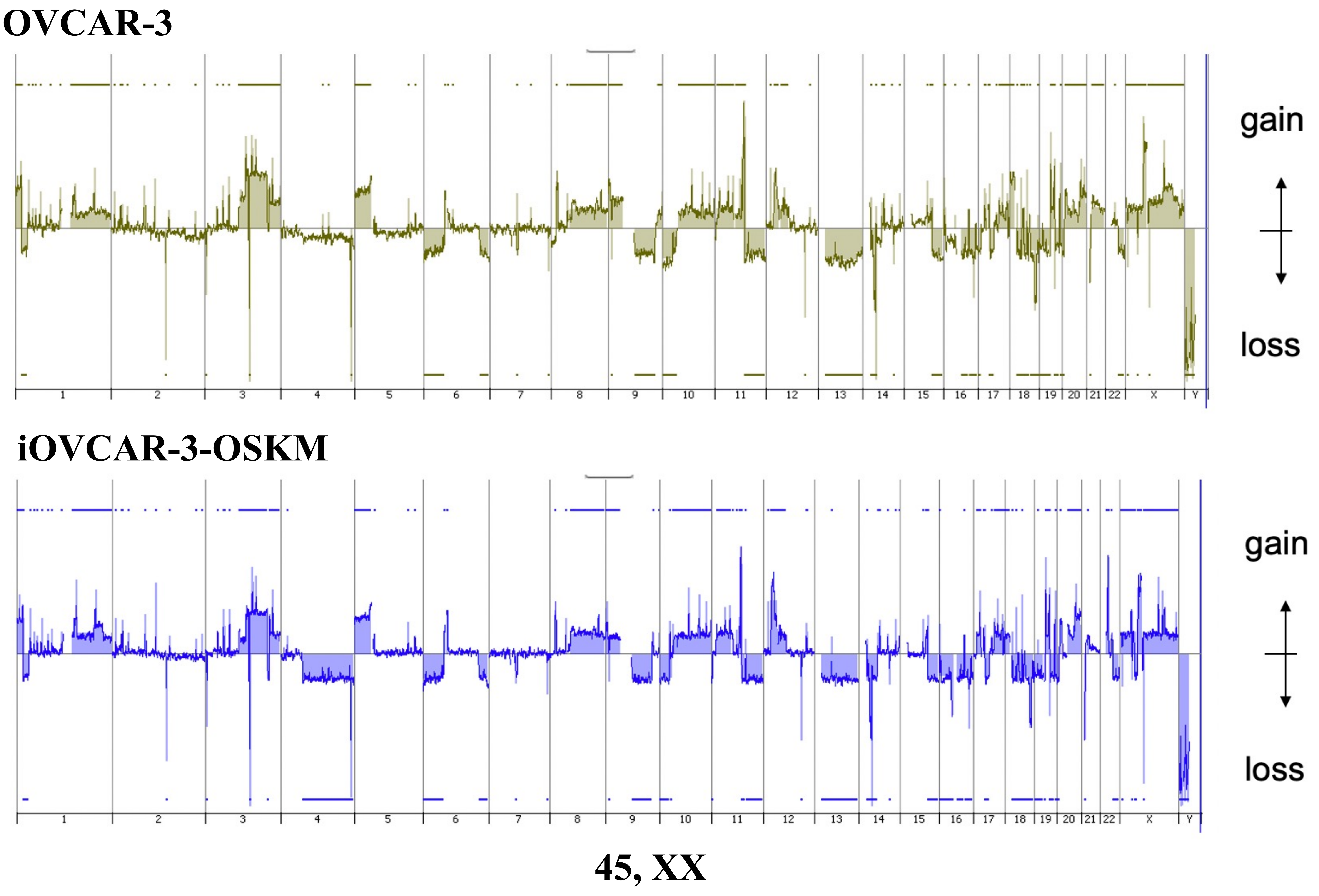

Figure S11. Array Comparative Genomic Hybridization (aCGH). aCGH analysis confirmed a 45, XX karyotype of OVCAR-3 and iOVCAR-OSKM, and the consistent detectable genetic alteration.
