# Supplementary material for: An experimental model for ovarian cancer: propagation of ovarian cancer initiating cells and generation of ovarian cancer organoids
Source: BMC Cancer. 2022 Sep 10;22:967. doi: 10.1186/s12885-022-10042-3 (PMC9463800; doi:10.1186/s12885-022-10042-3)
Supplement: Supplementary file 12 — Additional file 12: Figure S11. Uncropped gel of Figure S8B. Pluripotentgene expression and SeV silencing in iCA5171-OSKM clones. Expression of endogenouspluripotent genes (OCT4, SOX2, KLF4, and NANOG), SeV, and the housekeeping gene GAPDH. Neg Ctl: negative control (PCR mixture without cDNA). [file 12885_2022_10042_MOESM12_ESM.pdf]

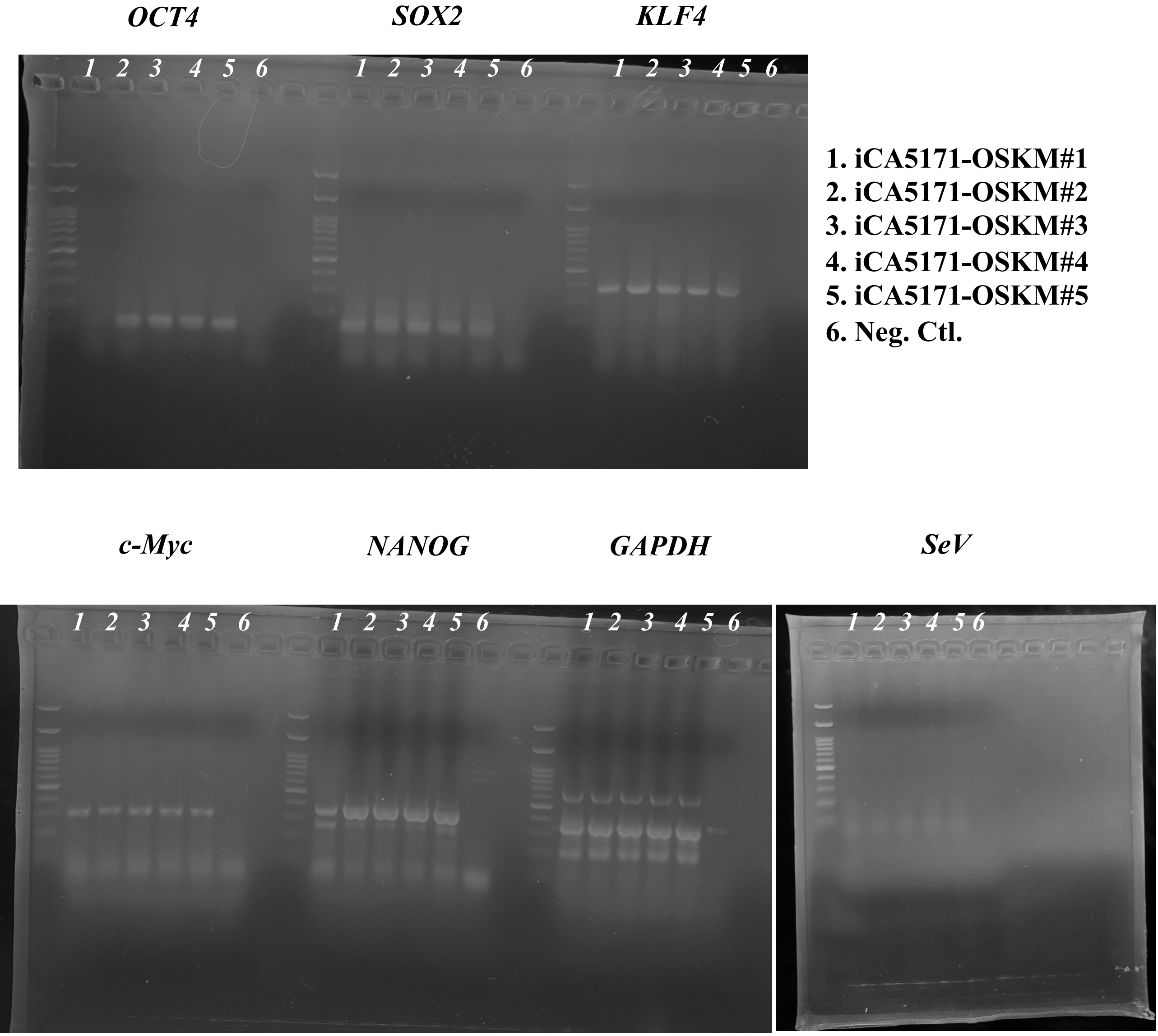

Figure S11. Uncropped gel of Figure S8B. Pluripotent gene expression and SeV silencing in iCA5171-OSKM clones. Expression of endogenous pluripotent genes (*OCT4, SOX2, KLF4, and NANOG*), *SeV*, and the housekeeping gene *GAPDH*. Neg Ctl: negative control (PCR mixture without cDNA).
