# Supplementary material for: An experimental model for ovarian cancer: propagation of ovarian cancer initiating cells and generation of ovarian cancer organoids
Source: BMC Cancer. 2022 Sep 10;22:967. doi: 10.1186/s12885-022-10042-3 (PMC9463800; doi:10.1186/s12885-022-10042-3)
Supplement: Supplementary file 13 — Additional file 13: Figure S12. Expression of pluripotent genes and the correlation with OS in an EOC tissue array. (A) Correlation of each single marker with OS in patients with EOC. Statistical analysis was carried out using SPSS 22.0 (IBM Corp., Armonk, NY, USA). Kaplan–Meier curves were used to evaluate the correlation of highly expressed markers with OS. Comparisons of two groups were made by the log-rank test. *P < 0.05 was considered significant. (B) The correlation of the numbers of highly expressed markers with the OS in patients with EOC. Kaplan–Meier curves were used to evaluate the correlation of the number of highly expressed markers with OS. Comparisons of two groups were made by the log-rank test. *P < 0.05 was considered to indicate significance. [file 12885_2022_10042_MOESM13_ESM.pdf]

A EOC tissue array(n=18)

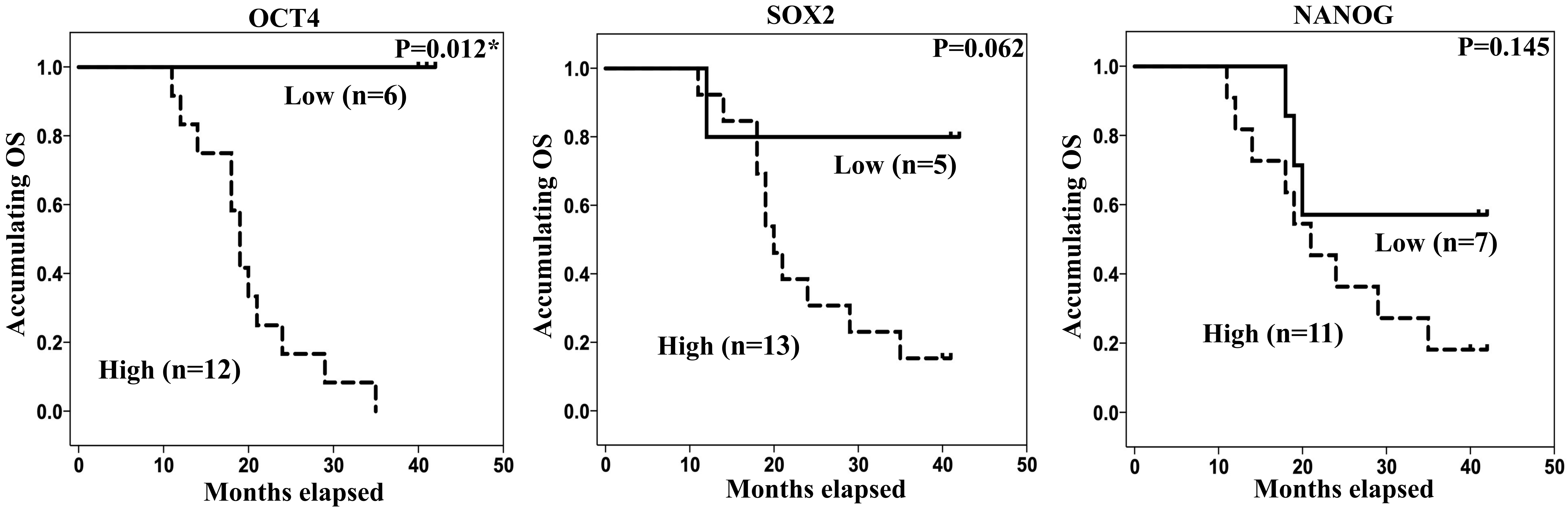

B EOC tissue array(n=18)

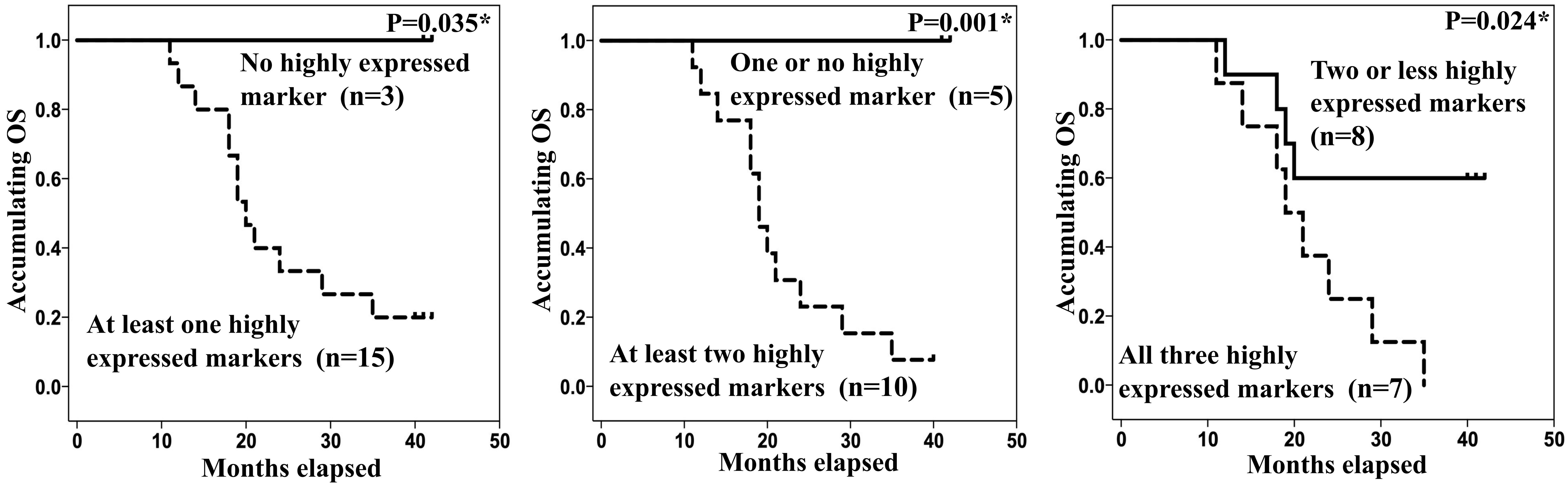

Figure S12. Expression of pluripotent genes and the correlation with OS in an EOC tissue array. (A) Correlation of each single marker with OS in patients with EOC. Statistical analysis was carried out using SPSS 22.0 (IBM Corp., Armonk, NY, USA). Kaplan–Meier curves were used to evaluate the correlation of highly expressed markers with OS. Comparisons of two groups were made by the log-rank test. *$P < 0.05$ was considered significant. **(B)** The correlation of the numbers of highly expressed markers with the OS in patients with EOC. Kaplan–Meier curves were used to evaluate the correlation of the number of highly expressed markers with OS. Comparisons of two groups were made by the log-rank test. *$P < 0.05$ was considered to indicate significance.
