# Supplementary material for: An experimental model for ovarian cancer: propagation of ovarian cancer initiating cells and generation of ovarian cancer organoids
Source: BMC Cancer. 2022 Sep 10;22:967. doi: 10.1186/s12885-022-10042-3 (PMC9463800; doi:10.1186/s12885-022-10042-3)
Supplement: Supplementary file 14 — Additional file 14: Figure S13. Expression of pluripotent genes and the correlation with OS in HGSOC cases of an EOC tissue array. (A) Correlation of each single marker with OS in eight patients with HGSOC. Statistical analysis was carried out using SPSS 22.0 (IBM Corp., Armonk, NY, USA). Kaplan–Meier curves were used to evaluate the correlation of highly expressed markers with OS. Comparisons of two groups were made by the log-rank test. *P < 0.05 was considered significant. (B) The correlation of the numbers of highly expressed markers with the OS in eight patients with HGSOC. Kaplan–Meier curves were used to evaluate the correlation of the number of highly expressed markers with OS. Comparisons of two groups were made by the log-rank test. *P < 0.05 was considered to indicate significance. [file 12885_2022_10042_MOESM14_ESM.pdf]

A HGSOC tissue array(n=8)

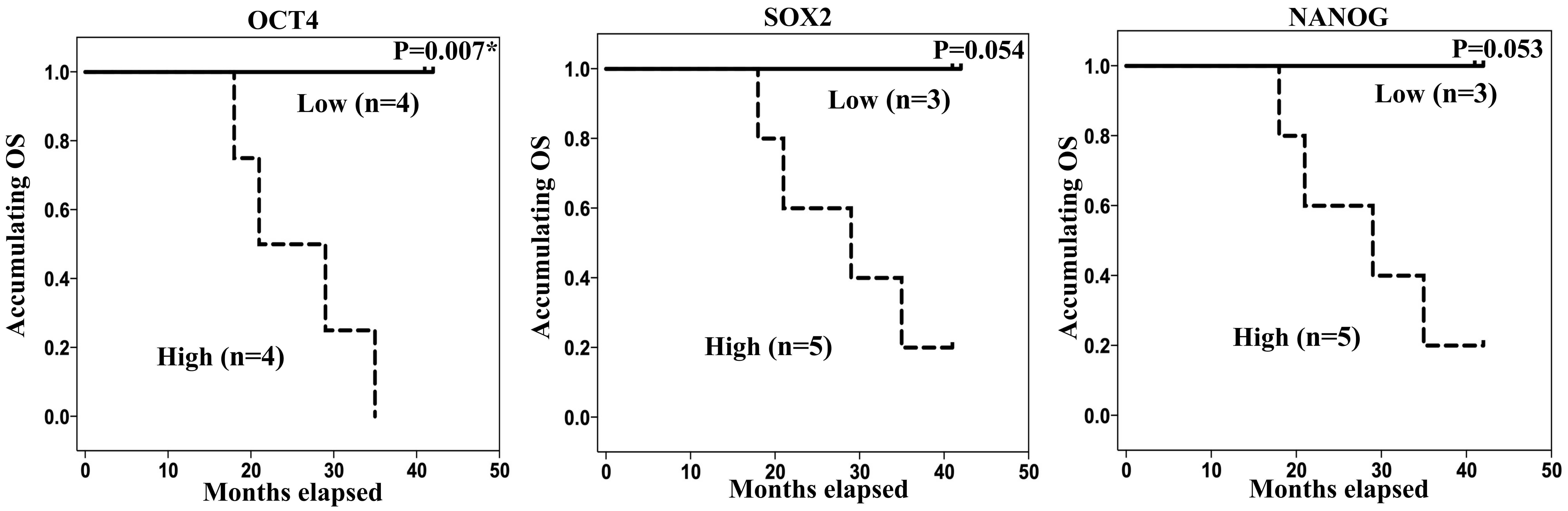

B HGSOC tissue array(n=8)

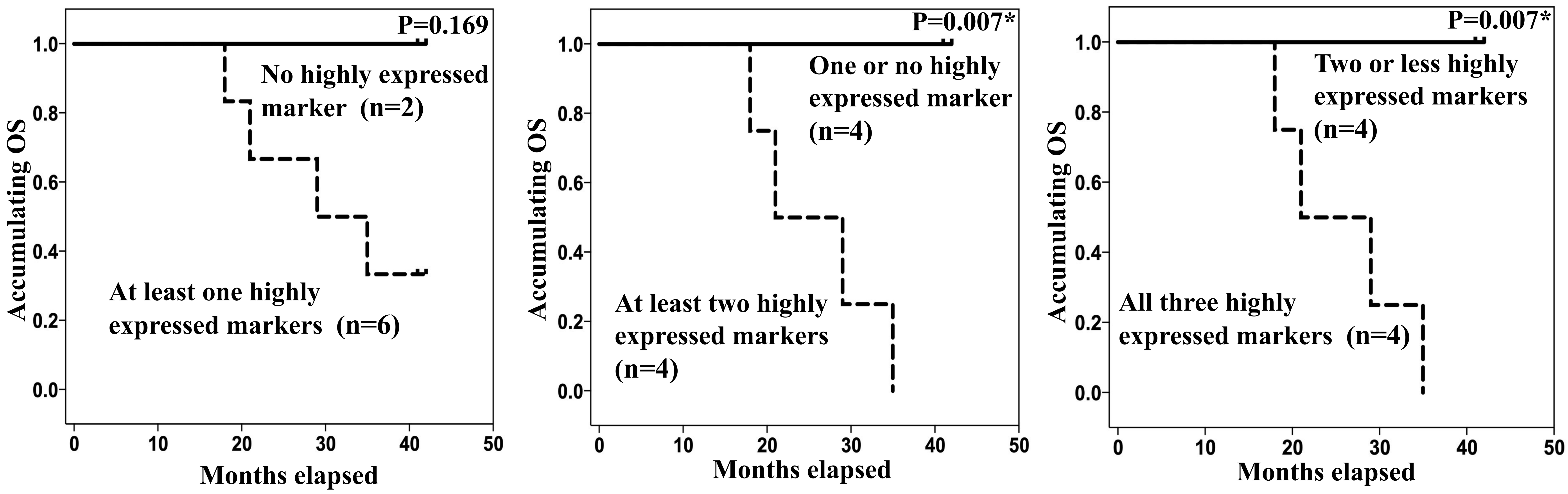

Figure S13. Expression of pluripotent genes and the correlation with OS in HGSOC cases of an EOC tissue array. (A) Correlation of each single marker with OS in eight patients with HGSOC. Statistical analysis was carried out using SPSS 22.0 (IBM Corp., Armonk, NY, USA). Kaplan–Meier curves were used to evaluate the correlation of highly expressed markers with OS. Comparisons of two groups were made by the log-rank test. *$P < 0.05$ was considered significant. **(B)** The correlation of the numbers of highly expressed markers with the OS in eight patients with HGSOC. Kaplan–Meier curves were used to evaluate the correlation of the number of highly expressed markers with OS. Comparisons of two groups were made by the log-rank test. *$P < 0.05$ was considered to indicate significance.
