# Supplementary material for: An experimental model for ovarian cancer: propagation of ovarian cancer initiating cells and generation of ovarian cancer organoids
Source: BMC Cancer. 2022 Sep 10;22:967. doi: 10.1186/s12885-022-10042-3 (PMC9463800; doi:10.1186/s12885-022-10042-3)
Supplement: Supplementary file 9 — Additional file 9: Figure S8. Establishment of the iCA5171-OSKM model, which exhibited stemness and cancer-initiating cell properties. (A)Schematic of the transduction procedure and establishment of iOCICs. CA5171 cells were infectedwith SeV containing four factors (OSKM) at day 0 and replated onto MEF dishes. Tra-1-60 positive colonies were picked up on day 21 and day 28. Scale bars: 200 µm. (B)Pluripotent gene expression and SeV silencing in iCA5171-OSKM clones. Expression of endogenous pluripotent genes (OCT4, SOX2, KLF4, and NANOG), SeV,and the housekeeping gene GAPDH. NC: negative control (PCR mixture without cDNA). The samples derive from the same experiment and gels were processed in parallel. (C)Protein expression of OCT4, TRA1-60, NANOG, and CD133 in day 7 iCA5171-OSKM colonies. Colonies were cultured on feeder cells for 7 days. IF was performed with OCT4, TRA1-60, NANOG, and CD133 primary antibodies. Scale bars: 50 µm. (D) IF staining was performed with CK7 and CA125 antibodies under differentiation conditions in iCA5171-OSKM cells. Scale bars: 50 µm. (E) Sphere-formation ability in vitro. A total of 105cells were plated on low attachment dishes and cultured for 14 days. The numbers of spheroids (≥ 25 µm) were counted under a microscope. The error bars indicate the SD. Scale bars: 200 µm. [file 12885_2022_10042_MOESM9_ESM.pdf]

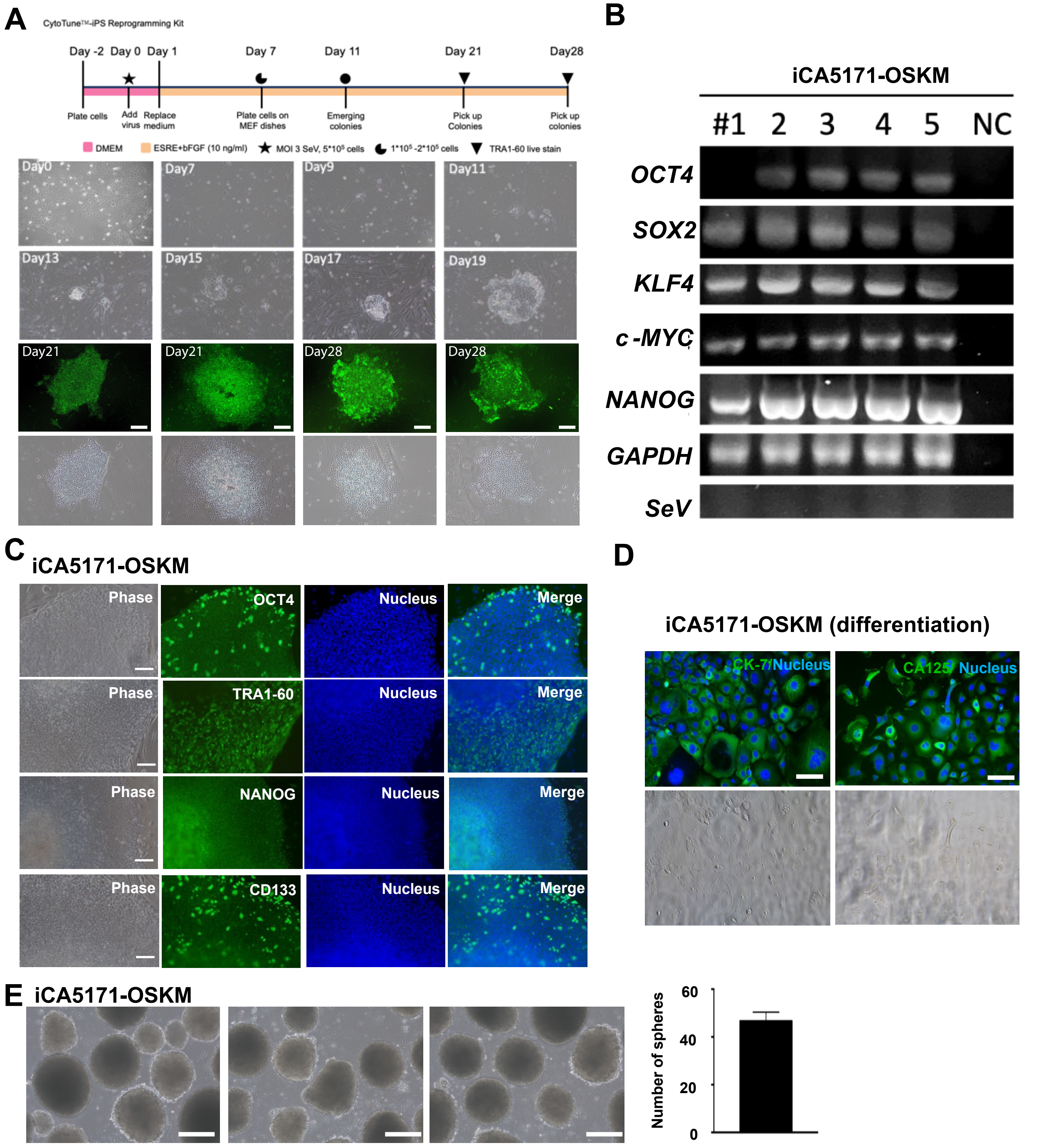

Figure S8. Establishment of the iCA5171-OSKM model, which exhibited stemness and cancer-initiating cell properties. (A) Schematic of the transduction procedure and establishment of iOCICs. CA5171 cells were infected with SeV containing four factors (OSKM) at day 0 and replated onto MEF dishes. Tra-1-60 positive colonies were picked up on day 21 and day 28. Scale bars: 200 µm. **(B)** Pluripotent gene expression and SeV silencing in iCA5171-OSKM clones. Expression of endogenous pluripotent genes (*OCT4, SOX2, KLF4*, and *NANOG*), *SeV*, and the housekeeping gene *GAPDH*. NC: negative control (PCR mixture without cDNA). The samples derive from the same experiment and gels were processed in parallel. **(C)** Protein expression of OCT4, TRA1-60, NANOG, and CD133 in day 7 iCA5171-OSKM colonies. Colonies were cultured on feeder cells for 7 days. Scale bars: 50 µm. **(D)** IF staining was performed with CK7 and CA125 antibodies under differentiation conditions in iCA5171-OSKM cells. Scale bars: 50 µm. **(E)** Sphere-formation ability in vitro. A total of 10^5 cells were plated on low attachment dishes and cultured for 14 days. The numbers of spheroids (≥ 25 µm) were counted under a microscope.The error bars indicate the SD. Scale bars: 200 µm.
